# Phytochemical Analysis, Antioxidant Activity, and Inhibition of Digestive Enzymes of *Carica papaya* L. Leaf

**DOI:** 10.3390/molecules31132394

**Published:** 2026-07-07

**Authors:** Juan Daniel Cruz-Castillo, Manasés González-Cortazar, Paulina Hernández-Hernández, Alejandro Zamilpa, Ana Silvia Gutiérrez-Román, Abraham Gómez-Rivera, Ricardo López-Rodríguez, David Ruiz-Ramos, German Alberto Nolasco-Rosales, Carlos Alfonso Tovilla-Zárate, Isela Esther Juárez-Rojop

**Affiliations:** 1 División Académica de Ciencias de la Salud, Universidad Juárez Autónoma de Tabasco, Villahermosa 86100, Tabasco, Mexico; 241e62003@alumno.ujat.mx (J.D.C.-C.); david.ruiz@ujat.mx (D.R.-R.); german.nolasco@ujat.mx (G.A.N.-R.); 2 Centro de Investigación Biomédica del Sur, Instituto Mexicano del Seguro Social, Xochitepec 62790, Morelos, Mexico; paulina.hernandez@uaem.edu.mx (P.H.-H.); alejandro.zamilpa@imss.gob.mx (A.Z.); 3 Centro de Desarrollo de Productos Bióticos, Instituto Politécnico Nacional (IPN), Carretera Yautepec-Jojutla, Km 6, Col. San Isidro, Yautepec 62731, Morelos, Mexico; agutierrezr1501@alumno.ipn.mx; 4 División Académica de Ciencias Básicas, Universidad Juárez Autónoma de Tabasco, Carretera Cunduacán-Jalpa Km 1, Col. La Esperanza, Cunduacán 86690, Tabasco, Mexico; abraham.gomez@ujat.mx (A.G.-R.); ricardo.lopezr@ujat.mx (R.L.-R.); 5 División Académica Multidisciplinaria de Comalcalco, Universidad Juárez Autónoma de Tabasco, Comalcalco 86650, Tabasco, Mexico; carlos.tovilla@ujat.mx

**Keywords:** *Carica papaya*, antioxidant, α-glucosidase, pancreatic lipase, clitorin

## Abstract

Medicinal plants are being investigated as a source of compounds with biological activities related to diabetes. The antidiabetic properties of the plant *Carica papaya* have been reported in experimental models. This study aimed to evaluate the phytochemical composition, antioxidant activity, and inhibitory activity of extracts from *C. papaya* leaves against α-glucosidase and pancreatic lipase. Plant material was collected in Tabasco, Mexico, and extracted by sequential maceration with solvents of increasing polarity: hexane, dichloromethane, methanol, and methanol:water. The extracts were fractionated by column chromatography, and the most active fractions were selected for further purification. The phytochemical identification of the active compounds was performed, and their structures were elucidated using spectroscopic and spectrometric techniques. The methanolic extract, rich in phenols and flavonoids, showed the highest antioxidant capacity (DPPH: 8.99 mmol TE/g; ABTS: 35.94 mmol RE/g; FRAP: 48.62 mmol Fe^2+^/g). The hydroalcoholic extract exhibited α-glucosidase inhibitory activity (38.44%), and bioassay-guided fractionation led to the identification of clitorin. The dichloromethane extract showed pancreatic lipase inhibition (52.2%), and the most active fraction contained loliolide. These findings demonstrate that *C. papaya* leaves contain bioactive compounds with antioxidants and digestive enzyme inhibitory activities, suggesting they could be candidates for further research in the management of diabetes.

## 1. Introduction

Diabetes is a group of conditions related to carbohydrate metabolism, characterized by inefficient glucose utilization and excessive glucose production, resulting in hyperglycemia [1]. The development of diabetic complications is significantly influenced by chronic hyperglycemia, which is attributed to metabolic, inflammatory, and hemodynamic mechanisms [2]. The management of diabetes requires reaching optimal glycemic control with lifestyle modifications, followed by the administration of glucose-lowering medications [3]. The early initiation of pharmacological treatment is associated with improved glucose control and reduced long-term complications in diabetes. Pharmacological approaches to the treatment of diabetes include dipeptidyl peptidase IV inhibitors, biguanides, GLP-1 receptor agonists, selective sodium–glucose cotransporter type 2 inhibitors, sulfonylureas, thiazolidinediones, α-glucosidase inhibitors, meglitinide derivatives, and insulin [4].

Digestive enzymes are pharmacological targets for controlling postprandial hyperglycemia in diabetes. Specifically, inhibition of α-glucosidase activity diminishes glucose absorption in the gut, lowers the postprandial blood glucose peak, and helps regulate its fluctuations [5]. Acarbose, miglitol, and voglibose are used in clinical practice as inhibitors of this enzyme for the treatment of diabetes. However, these drugs present adverse gastrointestinal effects, such as abdominal distension and inflammation [6].

Evidence suggests that diabetes is associated with obesity, particularly with a high proportion of intra-abdominal fat, considered the most important factor in its development. Furthermore, high levels of glucose and free fatty acids can impair insulin action and the integrity of the pancreatic islets [7]. Obesity is characterized by adipose tissue dysfunction, leading to lipotoxicity in the liver and pancreas, creating an environment that favors diabetes [8]. Among the pharmacological options for managing diabetes and obesity is the inhibition of lipase, a digestive enzyme that catalyzes the hydrolysis of triacylglycerols into free fatty acids. There are drugs that inhibit the activity of this enzyme, thereby reducing fatty acid absorption [9]. Orlistat is a gastric and pancreatic lipase inhibitor used to reduce the absorption of dietary fats, thereby decreasing calorie intake [10]. This drug has been reported to reduce total and LDL cholesterol [11]. However, it can interfere with the absorption of fat-soluble vitamins and cause gastrointestinal side effects [10].

Herbal medicine has been explored as a source of compounds for the prevention and management of chronic disorders, including diabetes, because plants produce a range of secondary metabolites (alkaloids, terpenes, and phenols) with biological activities that have been evaluated in experimental studies [12]. Bioactive compounds derived from plants are considered promising sources for drug discovery, offering safer and potentially sustainable solutions for disease prevention and management [13]. A study demonstrated that three Mexican medicinal plants (*Ludwigia octovalvis*, *Cnidoscolus aconitifolius*, and *Crotalaria longirostrata*) inhibit digestive enzymes, among them α-amylase, α-glucosidase, and lipase [14].

The plant *Carica papaya*, native to southern Mexico and distributed throughout tropical and subtropical regions worldwide, produces fruit year-round and has perennial leaves [15]. This plant species contains a multitude of bioactive compounds, such as alkaloids, flavonoids, and lycopene, which contribute to its medicinal properties. The leaves, seeds, and fruit of the plant contain chemical compounds with antioxidant, immunomodulatory, and anti-inflammatory actions [16]. *C. papaya* leaves were evaluated in mouse models of diabetes, and they showed reductions in high fasting blood glucose and serum lipid levels [17]. Also, papaya leaves have exhibited antioxidant activity in vitro, as evidenced by increased glutathione levels and reduced reactive oxygen species and lipid peroxidation [18].

Despite the interest in the antidiabetic properties of *C. papaya*, the scientific literature indicates that few experimental studies have confirmed its effectiveness and identified the compounds responsible for this biological activity [19]. In this regard, it is necessary to further study the antidiabetic activity of *C. papaya* leaves, isolate the compounds responsible for the observed effects, and structurally characterize them. We hypothesized that *C. papaya* leaf extracts would contain bioactive compounds that inhibit α-glucosidase and pancreatic lipase and demonstrate antioxidant activity. Therefore, this study aimed to evaluate the in vitro inhibitory activity of *C. papaya* leaf extracts against α-glucosidase and pancreatic lipase, assess their antioxidant activity, and identify the bioactive compounds responsible for these activities using column-chromatography-based fractionation.

## 2. Results

### 2.1. Total Phenolic, Flavonoid, and Terpene Content of Extracts

The total phenolic, flavonoid, and terpene content of *C. papaya* extracts is shown in Table 1. The total content ranged from 11.42 ± 1.66 to 254.10 ± 6.83 GAE/g extract. The methanol extract (CPME) exhibited the highest phenolic content (254.10 ± 6.83 mg GAE/g extract), with no significant differences between them (*p* > 0.05). In contrast, dichloromethane extract (CPDM) contained significantly lower levels of phenolic compounds (47.05 ± 2.60 mg GAE/g extract), while hexane extract (CPHX) showed the lowest concentration (11.42 ± 1.66 mg GAE/g extract). Similarly, flavonoids were more abundant in the polar extracts, with the hydroalcoholic extract (CPHA) showing the highest content (175.66 ± 23.41 mg RE/g), followed by CPME and CPDM (139.41 ± 38.87 mg RE/g). In contrast, no flavonoids were detected in the CPHX extract. In contrast, the terpene content showed the opposite trend, increasing as solvent polarity decreased. The highest terpene concentration was found in CPHX (51.47 ± 6.47 mg ME/g extract), followed by CPDM (39.08 ± 7.27 mg ME/g), whereas CPHA showed the lowest value (2.34 ± 0.56 mg ME/g extract). Overall, polar extracts were enriched in phenolic compounds and flavonoids, while non-polar extracts contained higher levels of terpenoid compounds.

### 2.2. Antioxidant Activity

Antioxidant properties of *C. papaya* leaf extracts were evaluated using three complementary assays: DPPH, ABTS, and FRAP (Table 2). In the DPPH assay, CPME exhibited the highest radical scavenging activity, with a value of 8.99 ± 0.51 mmol TE/g extract, followed by CPDM (6.55 ± 0.57 mmol TE/g), CPHX (6.40 ± 0.33 mmol TE/g), and CPHA (5.99 ± 0.49 mmol TE/g). Similarly, in the ABTS assay, CPME exhibited the highest antioxidant activity (35.94 ± 0.28 mmol RE/g), comparable to CPHA (34.47 ± 3.66 mmol RE/g). Lower antioxidant activities were recorded for CPDM and CPHX, with values of 29.94 ± 1.43 and 21.72 ± 3.65 mmol RE/g, respectively. Notably, CPME had a superior reducing capacity in the FRAP assay, measuring 48.62 ± 1.72 mmol Fe^2+^/g, and CPHA showed a lower capacity of 6.34 ± 1.56 mmol Fe^2+^/g.

### 2.3. α-Glucosidase Inhibition

The CPHA extract showed the highest α-glucosidase inhibitory activity in vitro, with an inhibition value of 38.44 ± 5.94% (Table 3), compared with the other extracts. However, all extracts showed significantly lower inhibitory activity than the controls: acarbose (0.025 mg/mL) and *Camellia sinensis* (0.125 mg/mL), which exhibited 47.82 ± 1.67% and 57.32 ± 2.86% inhibition, respectively.

A series of bioactivity-guided fractionations was conducted to obtain further active fractions from CPHA (Table 4). In the first fractionation, CPC7-R1 showed the highest inhibition percentage (32.74 ± 6.87%) among the fractions and was therefore selected for sub-fractionation. This yielded the CPC8-R10 fraction, which showed a higher inhibition percentage (34.37 ± 3.11%) than the other fractions evaluated. Fractionation of CPC8-R10 produced the fraction CPC10-R12 (32.72 ± 3.81%), which, after further refraction, resulted in the purification of the chemical compound present in CPC11-R12, which showed an inhibition of 47.23 ± 9.62%, similar to the acarbose control (47.82 ± 1.67%), and, consequently, was selected for enzyme kinetic investigations and chemical identification.

### 2.4. Phytochemical Composition of the Hydroalcoholic Extract of C. papaya

The CPHA extract was the most active in the α-glucosidase inhibition assays. Therefore, its chemical composition and that of its fractions were analyzed by high-performance liquid chromatography coupled with a photodiode array detector (HPLC-PDA), comparing retention times (*t*_R_) and UV spectra (λ_max_) with reference standards. CPHA showed the presence of quercetin glycoside, caffeic acid, kaempferol glycoside, and coumarins (Table 5). Through bioassay-guided fractionation (see Section 4), the active fraction CPC7-R1 was obtained, which contained myricetin tetraglycoside, rutin, ferulic acid, kaempferol glycoside, tiliroside, and kaempferitrin. Subsequent fractions produced from CPC7-R1 were also studied, including CPC8-R10, CPC10-R12, and CPC11-R12. CPC8-R10 contained rutin, glycosides of kaempferol and quercetin, as well as a caffeic acid derivative. CPC10-R12 contained rutin, kaempferol glycoside, a caffeic acid derivative, and tiliroside. Finally, CPC11-R12 showed a single major peak corresponding to a kaempferol glycoside, later identified as clitorin (94% purity) in this study.

### 2.5. Identification of Clitorin by Liquid Chromatography–Tandem Mass Spectrometry Analysis

**Figure 1:**
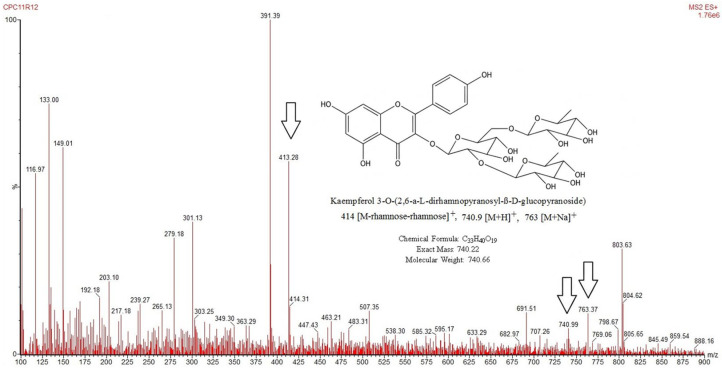
UPLC-MS/MS spectrum of the compound detected in the CPC11R12 fraction. The arrows indicate the diagnostic ions used for compound identification: the characteristic fragment ion at *m*/*z* 413.28, the protonated molecular ion [M + H]^+^ at *m*/*z* 740.9, and the sodium adduct [M + Na]^+^ at *m*/*z* 763.37. Red text indicates instrument software annotations.

The CPC11-R12 fraction was analyzed by ultra-high-performance liquid chromatography–tandem mass spectrometry (UPLC-MS/MS), which showed a protonated molecular ion peak [M + H]^+^ at *m*/*z* 740.99 and a fragment ion at *m*/*z* 414.31, indicating the presence of carbohydrate moieties linked to the flavonoid structure (Figure 1). Based on this fragmentation pattern, the chemical compound was tentatively identified as clitorin (kaempferol 3-O-(2,6-di-rhamnopyranosyl)-β-D-glucopyranoside; 94% purity) [20].

### 2.6. Determination of IC_50_ and Kinetic Analysis of α-Glucosidase Inhibition

**Figure 2:**
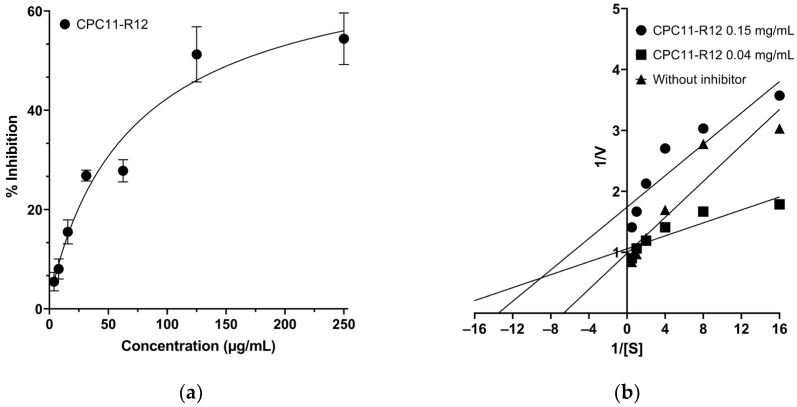
(**a**) Plot of enzyme inhibition percentage as a function of CPC11-R12 concentration; (**b**) Lineweaver–Burk plot of α-glucosidase in the absence and presence of CPC11-R12.

The analysis of CPC11-R12 demonstrated that its inhibitory activity is concentration-dependent, with an IC_50_ of 75 µg/mL (Figure 2a). In addition, to determine the mechanism of inhibition, we calculated the kinetic parameters of α-glucosidase. The enzyme without inhibitor showed a V_max_ of 1.454 µmol/min and a K_m_ of 0.438 mg/mL (Figure 2b). For CPC11-R12 at a concentration of 0.15 mg/mL (2 × IC_50_), the V_max_ was 0.702 µmol/min, and the K_m_ was 0.160 mg/mL. Furthermore, at a concentration of 0.04 mg/mL (½IC_50_), CPC11-R12 resulted in a V_max_ of 1.043 µmol/min and a K_m_ of 0.081 mg/mL (Figure 2b). These results are consistent with mixed-type inhibition [21].

### 2.7. Pancreatic Lipase Inhibition

The in vitro evaluation of *C. papaya* extracts demonstrated that CPDM exhibited the highest inhibitory activity against lipases (52.2 ± 9.74%) compared with other extracts (Table 6). Interestingly, this result was similar to that observed for the drug orlistat (48.03 ± 7.12%) and *C. sinensis* (53.51 ± 6.24%) inhibition. In contrast, the CPME, CPHX, and CPHA extracts showed considerably lower inhibition compared with the controls (*p* ≤ 0.001). Based on the CPDM inhibition results, it was fractionated. The CPC1-R2, CPC1-R9, and CPC1-R11 fractions, obtained from CPDM, showed inhibition percentages (83.33 ± 4.28%, 75.52 ± 5.96%, and 78.03 ± 9.62%, respectively) that were significantly higher than those of orlistat and *C. sinensis* (*p* ≤ 0.001). The CPC1-R11 fraction was selected for subsequent sub-fractionation, with CPC6-R9 being the most active, achieving 94.23 ± 5.0% inhibition. Due to its high inhibitory activity, CPC6-R9 was selected for chemical identification, IC_50_ determination, and subsequent kinetic analysis.

### 2.8. Gas Chromatography–Mass Spectrometry Characterization

Gas chromatography–mass spectrometry (GC-MS) analysis of the CPDM showed two major peaks corresponding to 3 possible compounds (two co-eluting). The chromatogram is shown in the Supplementary Material (Figure S11), and Table 7 presents the *t_R_*, peak area, compound names, and match factor (MF). The CPDM was found to contain the following compounds: cyclopropanebutanoic acid derivative (*t_R_* 17.07 min; 55.33%, MF 719) and two co-eluting compounds at *t_R_* 18.70 min (44.67%): benz[e]azulene-3,8-dione derivative (MF 668) and 2-bromotetradecanoic acid (MF 653). Furthermore, the fraction CPC6-R9 obtained from CPDM showed a single peak, which was identified by gas mass spectrometry and ^1^H and ^13^C NMR as 6-hydroxy-4,4,7a-trimethyl-5,6,7,7a-tetrahydro-1-benzofuran-2(4H)-one (*t_R_* 15.79 min; 100%; MF 854), also known as loliolide (66% purity).

6-hydroxy-4,4,7a-trimethyl-5,6,7,7a-tetrahydro-1-benzofuran-2(4H)-one (Loliolide, 66% purity): ^1^H-NMR (CDCl_3_, see Supplementary Data) δ 5.66(s, H-5), 4.29 (dq, 10.3, 6.8, 5.2 Hz, H-6), 1.49 (dd, 14.5, 3.7 Hz, H-5α), 1.96 (dt, 14.5, 2.6 Hz, H-5β), 1.73 (d, br, 4.0 Hz, H-7α), 2.44 (dt, 13.8, 2.6 Hz, H-7β), 1.24 (s, 4α-Me), 1.43 (s, 4β-Me), 1.75 (s, 7α-Me); ^13^C-NMR (CDCl_3_, see Supplementary Materials) δ 182.85(C-3a), 172.21(C-2), 112.87 (C-3), 87.00 (C-7a), 66.70 (C-6), 47.29 (C-5), 47.65 (C-7), 33.95 (C-4), 30.72(4α-Me), 27.04 (7α-Me), 26.54 (4β-Me). The spectroscopic data were compared with those from the literature [22].

### 2.9. Determination of IC_50_ and Kinetic Analysis of Pancreatic Lipase Inhibition

**Figure 3:**
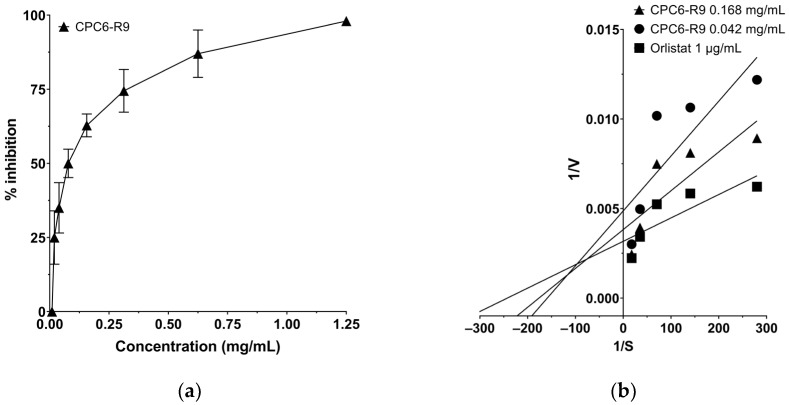
(**a**) Plot of enzyme inhibition percentage as a function of CPC6-R9 concentration; (**b**) Lineweaver–Burk plot of pancreatic lipase in the absence and presence of CPC6-R9.

The compound present in CPC6-R9 had an IC50 of 0.08392 mg/mL (95% CI: 0.06220–0.1132 mg/mL) in the nonlinear regression analysis of the dose–response curve (Figure 3a). CPC6-R9 was selected for kinetic analysis to determine the mechanism of inhibition. CPC6-R9 showed concentration-dependent inhibition. It reduced V_max_ (0.011 ΔAbs/min) and K_m_ (49.67 μM) at a concentration of 0.168 mg/mL (2 × IC_50_) compared to the inhibitor-free control (V_max_ = 0.040 ΔAbs/min; K_m_ = 115.50 μM). When CPC6-R9 was tested at 0.042 mg/mL (½IC_50_), the decrease was smaller, with values of V_max_ = 0.015 ΔAbs/min and K_m_ = 54.70 μM (Figure 3b). The reduction in both kinetic parameters with CPC6-R9 is consistent with mixed-type enzyme inhibition [21].

### 2.10. Predicted Absorption, Distribution, Metabolism, Excretion, and Toxic Properties

Predictive analysis of the pharmacokinetic properties of clitorin suggests limited intestinal absorption (Table 8), moderate plasma protein binding (82.0%), and a low probability of penetration across the blood–brain barrier. Furthermore, a plasma clearance of 0.98 mL/min/kg, a low probability of cardiotoxicity (hERG blockers: 0.01), and a low probability of CYP2C8 inhibition were expected. The loliolide assay suggests acceptable intestinal absorption (Caco-2 permeability) and considerable plasma protein binding (73.3%). Possible interactions with CYP3A4 and CYP2C19, plasma clearance of 7.40 mL/min/kg, and low probability of cardiotoxicity (hERG blockers: 0.05). Full absorption, distribution, metabolism, excretion, and toxic (ADMET) prediction results are available in the Supplementary Materials.

## 3. Discussion

Current research on herbal medicines has enabled the linking of chemical composition to biological activity [12]. In this context, the present study evaluated the inhibitory activity of digestive enzymes and the antioxidant activity of *C. papaya* leaf extracts to relate these activities to their phytochemical composition.

In the phytochemical composition of *C. papaya* extracts, CPME and CPHA had the highest levels of phenolic compounds and flavonoids, whereas CPDM and CPHX had significantly lower levels. In this context, compound extraction from *C. papaya* leaves is influenced by the type of solvent used; medium- and high-polarity solvents are more effective for saponins, phenols, and flavonoids [15]. The high total phenolic and flavonoid contents observed in CPHA were further supported by its HPLC-PDA profile, which revealed the presence of phenolic acids, coumarins, and flavonoid derivatives (quercetin and kaempferol glycosides). According to available data, *C. papaya* leaves contain several phenolic and flavonoid compounds, such as kaempferol, quercetin, rutin, catechin, gallic acid, and apigenin [23]. Our study showed that clitorin, a type of kaempferol glucoside, was isolated and identified from CPHA. In another investigation, it was reported that *C. papaya* contains clitorin and other glycosylated derivatives of kaempferol [20]. On the other hand, the total terpene content varied between the extracts, with the highest concentration observed in non-polar CPHX and CPDM. This behavior can be attributed to the predominantly lipophilic nature of terpenoids, which are more readily solubilized and extracted by low-polarity solvents such as hexane and dichloromethane [24]. CPDM contained mainly nonpolar chemical compounds, comprising brominated fatty acids, benzofuran derivatives, and cyclopropane molecules. Similar solvent-dependent extraction profiles have been reported for *C. papaya*, where non-polar fractions are enriched in terpenes, sterols, and triterpenes. In contrast, polar fractions are enriched in phenolic compounds and flavonoids [25,26].

The present study demonstrated that *C. papaya* leaf exhibits α-glucosidase inhibitory activity. Previous reports show that seeds of *C. papaya* exhibit inhibitory effects on carbohydrate-digesting enzymes [27], suggesting that this species contains bioactive metabolites of pharmacological relevance distributed throughout *C. papaya*. Notably, the highest α-glucosidase inhibitory activity was observed in CPHA and was associated with the presence of flavonoid glycosides. Flavonoids have been widely reported as effective α-glucosidase inhibitors, and their activity is strongly influenced by structural features such as a planar conjugated system and hydroxyl substitution patterns in the flavonoid scaffold [28]. In the current study, bioactivity-guided fractionation of CPHA revealed the presence of kaempferol derivatives in active fractions. Tiliroside, observed in the first fraction, is a kaempferol glycoside and has been reported as an α-glucosidase inhibitor in another plant, highlighting the relevance of this metabolite class for enzyme inhibition [29]. The successive purification led to the isolation of flavanol clitorin as the major compound in the most active fraction. Flavanols have been linked to α-glucosidase inhibitory activity in other plants, including *C. sinensis* [30]. Although prior research shows the antidiabetic effects of *C. papaya* leaf extracts, few studies have identified clitorin as one of its active compounds. Clitorin and other glycosylated kaempferol derivatives must have been associated with biological activities related to metabolic regulation [31]. Furthermore, computational studies using molecular docking have revealed that clitorin has a high binding affinity for protein targets, supported by its capacity to form multiple hydrogen-bond and hydrophobic interactions, which may favor its interaction with enzyme targets [32]. It is worth noting that clitorin exhibited partial α-glucosidase inhibitory activity. However, kaempferol derivatives have been reported to act synergistically with other compounds, thereby enhancing inhibition [33]. Similarly, individual phenolic compounds often show moderate activity, requiring higher concentrations to achieve significant effects [34]. The findings indicate that kaempferol glycosides, primarily clitorin, contribute to the α-glucosidase-inhibitory activity observed in *C. papaya* leaves.

Previously, it was reported that the methanol extract of *C. papaya* fruit peel exhibits pancreatic lipase inhibitory activity [31]; similarly, we found that the leaves possess this activity, particularly in the dichloromethane extract. Regarding the compounds identified in this crude extract, it has been previously described that one of these compounds with various biological activities is a cyclopropane derivative (cyclopropanebutanoic acid, 2-[[2-[[2-[(2-pentylcyclopropyl)methyl]cyclopropyl]methyl]cyclopropyl]methyl]-, methyl ester), which in other plants has been associated with antioxidant, antimicrobial, and anti-inflammatory properties [35,36]. On the other hand, a benzazulene derivative (Benz[e]azulene-3,8-dione, 3a,4,6a,7,9,10,10a,10b-octahydro-3a,10a-dihydroxy-5-(hydroxymethyl)-7-(1-hydroxy-1-methylethyl)-2,10-dimethyl-) was found in *Clitoria ternatea*, a plant recognized for its ability to inhibit digestive enzymes [37]. In addition, biodirected fractionation of pancreatic lipase inhibitory activity found the presence of 6-hydroxy-4,4,7α-trimethyl-5,6,7,7α-tetrahydro-1-benzofuran-2(4H)-one in the most active fraction. This compound has been identified as loliolide, a naturally occurring monoterpenoid lactone found in seaweeds and recognized for its neuroprotective and anti-inflammatory properties [22]. In an in vitro study, loliolide isolated from *Sargassum horneri* reduced lipid accumulation, suggesting its involvement in lipid metabolism [38]. Loliolide has already been found in *C. papaya* leaves [20]; however, evidence regarding its contribution to the biological activities of this species remains limited. The present study provides the first evidence that loliolide isolated from *C. papaya* leaves exhibits pancreatic lipase inhibitory activity.

The biological activity, bioavailability, and bioaccessibility of plant secondary metabolites are determined by their molecular structure, concentration, and digestion [39]. It is worth noting that neither clitorin nor loliolide has been sufficiently studied in the context of digestive enzyme inhibition. However, both compounds exhibited promising pharmacokinetic profiles, and their absorption behavior appears particularly relevant given their possible site of action in the gastrointestinal tract. Computational predictions revealed that clitorin has poor permeability across Caco-2 cells and exhibits negative intestinal absorption, suggesting that its α-glucosidase inhibitory activity may occur primarily in the intestinal lumen. In contrast, loliolide was positive for intestinal absorption and showed adequate permeability, suggesting it reaches the systemic circulation. In addition to a possible local effect on pancreatic lipase, loliolide may provide metabolic benefits, possibly through interactions with CYP3A4 and CYP2C19. Although these projections are not equivalent to experimental tests, they have been described as potentially useful for identifying pharmacokinetic issues that arise during drug development research [40]. Furthermore, molecular docking studies could provide valuable insights into the binding interactions of clitorin and loliolide with their respective enzyme targets (α-glucosidase and pancreatic lipase), helping to elucidate the structural basis of their inhibitory activity. Further research is necessary to prove the efficacy and safety of these compounds under physiological conditions.

The antioxidant assays demonstrated that CPME possessed the highest antioxidant activity, as evidenced by DPPH, ABTS, and FRAP results, which closely corresponded with its phytochemical content, indicating that phenolic compounds are major contributors to the antioxidant activity of *C. papaya* extracts through their ability to donate electrons or hydrogen atoms and neutralize reactive species [41,42]. It is interesting to mention that the CPHA extract exhibited lower DPPH and FRAP values than those observed for CPHX, despite its low content of phenols and flavonoids. This suggests that other metabolites, such as terpenoids, may contribute to the reducing power. Overall, our findings indicated that the antioxidant properties of *C. papaya* extracts arise from the combined action of different classes of secondary metabolites, with phenolic compounds being the main compounds with this activity.

In conclusion, our phytochemical analysis shows that the polarity of the extraction solvent notably influences the composition and biological activity of *C. papaya* leaf extracts. Specifically, CPME exhibited higher antioxidant capacity than the other extracts and a high content of phenols and flavonoids. CPHA was notable for its α-glucosidase inhibitory activity, and bioassay-guided fractionation yielded the compound clitorin. Furthermore, CPDM demonstrated significantly higher pancreatic lipase inhibitory activity and contained nonpolar compounds, among which loliolide was detected in its most active fraction. Further studies on the pharmacological properties of these compounds are still needed. This study provides novel evidence for clitorin and loliolide as digestive enzyme inhibitors from *C. papaya* leaves, contributing to the understanding of this species bioactive compounds.

## 4. Materials and Methods

### 4.1. Plant Material Collection and Preparation

The leaves of *C. papaya* were collected in Huapacal 1ra Sección, Jalpa de Méndez, Tabasco, Mexico (18°11′32.4″ N, 93°09′22.1″ W), in April 2024. The specimen was recognized and conserved with receipt number 037351 in the herbarium of the Academic Division of Biological Sciences of Universidad Juárez Autónoma de Tabasco (UJAT). The leaves were washed with water, air-dried at room temperature (25 ± 2 °C), and ground. Sequential maceration with solvents of increasing polarity was performed to obtain extracts containing different metabolite classes, as the solubility of plant compounds largely depends on solvent polarity [43]. A total of 4 kg of plant material was placed in a container with 4 L of solvent and macerated for 24 h. The extract was then filtered through Whatman No. 4 filter paper and concentrated to dryness on a rotary evaporator [44]. This procedure was carried out in triplicate with *n*-hexane (HPLC grade, Sigma-Aldrich, St. Louis, MO, USA), dichloromethane (HPLC grade, Sigma-Aldrich, St. Louis, MO, USA), methanol (HPLC grade, Sigma-Aldrich, St. Louis, MO, USA), and a hydroalcoholic mixture of methanol:water (50:50, *v*/*v*; methanol, HPLC grade, Sigma-Aldrich, St. Louis, MO, USA; ultrapure water, Milli-Q, Merck Millipore, Burlington, MA, USA). The extraction process yielded the hexane extract (CPHX; 180 g, 4.5% *w*/*w*), dichloromethane extract (CPDM; 144 g, 3.6% *w*/*w*), methanol extract (CPME; 87 g, 2.2% *w*/*w*), and hydroalcoholic extract (CPHA; 93 g, 2.3% *w*/*w*). In addition, a hydroalcoholic extract was prepared by maceration from commercial leaves of *C. sinensis* (ACH Foods Mexico, S. de R.L. de C.V., Mexico City, Mexico) for plant control in enzymatic assays due to its inhibitory activity against digestive enzymes [30].

### 4.2. Determination of Total Phenolic Content

The phenolic content of *C. papaya* leaf extracts was determined using the Folin–Ciocalteu method in a 96-well microplate, based on Singleton and Rossi [45] with slight modifications. This involved mixing 20 μL of diluted extracts (50 μg/mL) with 100 μL of Folin–Ciocalteu reagent (1:10, *v*/*v*; Sigma-Aldrich, St. Louis, MO, USA) for 60 s. After 5 min, 80 μL of a 10% *w*/*v* sodium carbonate solution was added, and the mixture was agitated for 1 min. The reaction mixture was incubated at ambient temperature for 30 min, and the absorbance was measured at 765 nm with a microplate reader (BioTek ELx800; BioTek Instruments Inc., Winooski, VT, USA). Gallic acid (≥98%, Sigma-Aldrich) dilutions (20–200 μg/mL) were used to construct the calibration curve (y = 0.0056x − 0.5007 R^2^ = 0.996) The total phenolic content was expressed as mg of Gallic Acid Equivalents (GAE) per gram of extract (mg GAE/g extract) and calculated according to the following Equation (1):

(1) $$\mathrm{TPC}\;\left(\frac{\mathrm{mg}\;\mathrm{GAE}}{g\;\mathrm{extract}}\right)=\frac{C_{\mathrm{GAE}\;}\;\times \;\mathrm{DF}\;\times \;V}{C_{\mathrm{extract}}}$$

where *C* is the Gallic Acid Equivalent concentration obtained from the calibration curve, *DF* is the dilution factor, *V* is the final volume of the reaction mixture (mL), and *C* is the concentration of the extract used in the assay (g/mL).

### 4.3. Determination of Total Flavonoid Content

Total flavonoid content of *C. papaya* leaf extracts was determined using the aluminum chloride method, adapted from Shraim et al. [46], with slight modifications. Rutin standard solutions (1–70 μg/mL; ≥95%, Sigma-Aldrich, St. Louis, MO, USA) were prepared in methanol (HPLC grade, Sigma-Aldrich). In a 96-well microplate, 20 μL of extracts (50 μg/mL) or rutin standard was mixed with 80 μL of distilled water and 10 μL of sodium acetate (5%; ≥99%, Sigma-Aldrich, St. Louis, MO, USA). After 5 min, 30 μL of aluminum chloride solution (10%; ≥98%, Sigma-Aldrich, St. Louis, MO, USA) and 60 μL of methanol were added (final volume 200 μL). This mixture was incubated at room temperature for 30 min, and absorbance was recorded at 450 nm (BioTek ELx800; BioTek Instruments Inc., Winnoski, VT, USA). Rutin dilutions (25–200 μg/mL) were used to construct the calibration curve (y = −0.0017x + 0.0548 R^2^ = 0.992). The total flavonoid content was expressed as mg of Rutin Equivalents (RE) per gram of extract (mg RE/g extract) and calculated according to the following Equation (2):

(2) $$\mathrm{TFC}\;\left(\frac{\mathrm{mg}\;\mathrm{RE}}{g\;\mathrm{extract}}\right)=\frac{C_{\mathrm{RE}\;}\;\times \;\mathrm{DF}\;\times \;V}{C_{\mathrm{extract}}}$$

where *C* is the Rutin Equivalent concentration obtained from the calibration curve, *DF* is the dilution factor, *V* is the final volume of the reaction mixture (mL), and *C* is the concentration of the extract used in the assay (g/mL).

### 4.4. Quantification of Total Terpene Content

In the assay to measure the total terpene concentration of *C. papaya* leaf extracts, a 200 μL sample was mixed with 1.5 mL of chloroform (≥99.8%, HPLC grade, Sigma-Aldrich, St. Louis, MO, USA) and allowed to stand for 3 min [47]. Subsequently, 100 μL of sulfuric acid (≥95%, Sigma-Aldrich, St. Louis, MO, USA) was added, and the mixture was allowed to precipitate and incubated in the dark for 2 h. The supernatant was carefully discarded, and the precipitate was thoroughly combined with methanol. The absorbance was then measured at 540 nm (BioTek ELx800; BioTek Instruments Inc., Winnoski, VT, USA). The calibration curve was established using menthol (≥99%, Sigma-Aldrich, St. Louis, MO, USA). Menthol dilutions (200–500 μg/mL) were used to construct the calibration curve (y = −0.0023x + 0.0851 R^2^ = 0.989). The total terpenoid content was expressed as mg of Menthol Equivalents (ME) per gram of extract (mg ME/g extract) and calculated according to the following Equation (3):

(3) $$\;\mathrm{TTC}\;\left(\frac{\mathrm{mg}\;\mathrm{ME}}{g\;\mathrm{extract}}\right)=\frac{C_{\mathrm{ME}\;}\;\times \;\mathrm{DF}\;\times \;V}{C_{\mathrm{extract}}}$$

where *C* is the Menthol Equivalent concentration obtained from the calibration curve; DF is the dilution factor; V is the final volume of the reaction mixture (mL); and C is the concentration of the extract used in the assay (g/mL).

### 4.5. Bioassay-Guided Fractionation

**Figure 4:**
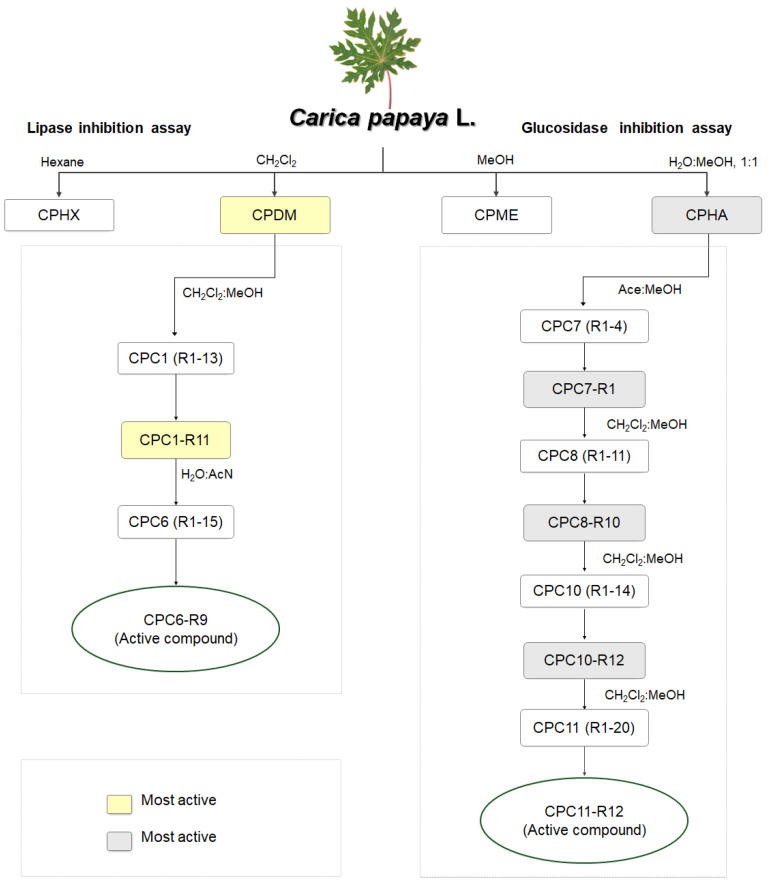
Bioassay-guided fractionation flowchart of *C. papaya* leaf extracts showing the selection of the most active fractions against lipase and α-glucosidase. Rectangles represent extracts and fractions obtained during the fractionation process. Yellow (lipase assay) and gray (α-glucosidase assay) rectangles indicate the most active fractions selected for further purification, while ovals denote the purified active compounds. Elution systems: CH_2_Cl_2_, dichloromethane; MeOH, methanol; H_2_O:MeOH, water:methanol; Ace:MeOH, acetone:methanol; CH_2_Cl_2_:MeOH, dichloromethane:methanol; H_2_O:AcN, water:acetonitrile.

The CPDM (144 g) showed the highest activity in the lipase assay and was selected for fractionation by normal-phase column chromatography (silica gel 60, Merck KGaA, Darmstadt, Germany; column 5 × 60 cm) using a dichloromethane: methanol gradient system (dichloromethane and methanol, HPLC grade, Sigma-Aldrich, St. Louis, MO, USA) with increasing polarity, yielding 13 fractions designated CPC1-R1 to R13 (Figure 4). Among these, fraction CPC1-R11 (7.7 g, 5.35% relative to the CPDM) exhibited the highest lipase inhibitory activity and was subsequently subjected to further fractionation by reversed-phase chromatography (C18 silica gel, Merck KGaA, Darmstadt, Germany; column 3 × 50 cm) eluting with a water:acetonitrile gradient (HPLC grade, Sigma-Aldrich, St. Louis, MO, USA), resulting in 15 fractions named CPC6-R1 to R15, with CPC6-R9 (42 mg, 0.55% relative to CPC1-R11) showing the highest lipase inhibitory activity. This fraction was examined using GC-MS, which revealed a single compound.

The CPHA extract (93 g) was the most active in the α-glucosidase assay. This extract was fractionated by normal-phase column chromatography (silica gel 60, Merck KGaA, Darmstadt, Germany; column 5 × 60 cm) using an acetone:methanol gradient system (HPLC grade, Sigma-Aldrich, St. Louis, MO, USA), yielding 4 fractions named CPC7-R1 to R4. The most active fraction, CPC7-R1 (6.0 g, 6.45% relative to CPHA), was subjected to further normal-phase column chromatography (silica gel 60, Merck KGaA, Darmstadt, Germany; column 3 × 50 cm) using a dichloromethane:methanol gradient system, affording 11 fractions designated CPC8-R1 to R11. Among these, CPC8-R10 (1.25 g, 20.83% relative to CPC7-R1) was the most active and was selected for an additional normal-phase fractionation (silica gel 60, Merck KGaA, Darmstadt, Germany; column 2.5 × 40 cm) using the same dichloromethane:methanol gradient system, yielding 14 fractions designated CPC10-R1 to R14. Fraction CPC10-R12 (573 mg, 45.84% relative to CPC8-R10) exhibited the highest activity and was further fractionated under the same conditions (column 2 × 30 cm), yielding 20 fractions designated CPC11-R1 to R20. Among these, CPC11-R12 (15.5 mg, 2.70% relative to CPC10-R12) was the most active in the α-glucosidase assay, and a pure compound was identified from this fraction.

All column fractions were collected every 50 mL. Fraction purity was evaluated by TLC (silica gel 60 F254, Merck KGaA, Darmstadt, Germany) and visualized under UV light (254 and 365 nm).

### 4.6. Determination of Phytochemicals

#### 4.6.1. HPLC-PDA Analysis Conditions

CPHA and its fractions were subjected to bioactivity-guided HPLC analysis to identify the active compound. The samples were analyzed using an HPLC system (Waters 2695 Separation Module, Waters, Milford, MA, USA) equipped with a diode array detector (UV detection range 200–600 nm). A Discovery^®^ C18 (5 μm) column (L × I.D. 25 cm × 4.6 mm; Supelco, Merck KGaA, Darmstadt, Germany) and the Pro-3 software (Version 3.8.1, Waters, Milford, MA, USA) were used. The mobile phase consisted of a gradient system with HPLC-grade H_2_O (Merck Millipore, Burlington, MA, USA) + trifluoroacetic acid (0.5%; Sigma-Aldrich, St. Louis, MO, USA) and acetonitrile (Merck KGaA, Darmstadt, Germany), over a 30 min elution time. The injection volume was 5 μL, and the flow rate was 0.9 mL/min. For peak identification, reference standards (quercetin 3-β-D-glucoside, kaempferol rhamnoside, rutin, caffeic acid, and p-coumaric acid; Sigma-Aldrich, St. Louis, MO, USA) were analyzed under the same chromatographic conditions. The chromatograms and UV-Vis spectra of all samples and standards are provided in the Supplementary Materials.

#### 4.6.2. UPLC-MS/MS Analysis Conditions

The fraction CPC11-R12 derived from CPHA extract was analyzed by mass spectrometry on an Acquity UPLC system coupled to a Xevo TQD triple quadrupole mass spectrometer (Waters Corp., Milford, MA, USA) equipped with an electrospray ionization (ESI, Zspray) source heated to 150 °C. The system comprised a quaternary pump, an autosampler with a photodiode array detector, and a column oven. The system was operated using Waters MassLynx v4.1 and Empower Chromatography Data System software (Waters Corp., Milford, MA, USA) suites for instrument control and data processing. Nitrogen served as the solvation gas at 700 L/h and 500 °C, while argon was used as the collision gas at 0.10 mL/min (Thermo Fisher Scientific, Bremen, Germany). Each sample (5 μL) was injected onto an Acquity UPLC BEH RP-18 column (2.1 mm × 50.0 mm, 1.7 μm; Waters Corp., Milford, MA, USA). The mobile phase consisted of a gradient system of water (Merck Millipore, Burlington, MA, USA) containing 0.5% trifluoroacetic acid (Sigma-Aldrich, St. Louis, MO, USA) and high-purity acetonitrile (Merck KGaA, Darmstadt, Germany) at a flow rate of 0.3 mL/min, with a total run time of 20 min.

#### 4.6.3. Conditions for GC-MS Analysis

The CPDM, being the most active against lipase, was chosen for purification, yielding the CPC6-R9 fraction, which was subsequently analyzed by GC-MS using an Agilent 7890B gas chromatograph coupled to an Agilent 7000D triple quadrupole mass selective detector (Agilent Technologies, Santa Clara, CA, USA). The Mass Hunter workstation (version 12.1) was used for data acquisition in electron ionization mode. Separation was performed on an HP-5 MS column (30 m × 250 μm × 0.25 μm) with a 5% diphenylmethylsiloxane coating. Helium (99.9%) served as the carrier gas at a constant flow rate of 1 mL/min. The injector and transfer line temperatures were set to 300 °C and 310 °C, respectively. The injection was carried out with a volume of 1 μL in splitless. mode. The oven temperature program was as follows: initial temperature of 50 °C held for 1 min, then ramped to 300 °C at 10 °C/min and held for 1 min, followed by a final ramp to 310 °C at 10 °C/min and held for 20 min. The total run time per sample was 49.78 min. Mass spectra were compared with the National Institute of Standards and Technology (NIST) database (version 14.0) for compound identification [48].

#### 4.6.4. NMR Data

^1^H and ^13^C NMR spectra were obtained on a Bruker AVANCE III HD 500 MHz (11.74 T) spectrometer (Bruker Corporation, Billerica, MA, USA) with a 1H/31P/15N-109Ag (5 mm) three-channel indirect radiofrequency probe. A 1H/15N-31P CPMAS multinuclear probe was used for 4 mm rotors and sample rotation speeds up to 15 kHz, with deuterated chloroform (99.8% D, Sigma-Aldrich, St. Louis, MO, USA) and tetramethylsilane (≥99.9%, Sigma-Aldrich, St. Louis, MO, USA) as the internal reference.

### 4.7. Antioxidant Assays

Antioxidant activity was measured using the following three methods.

#### 4.7.1. DPPH Radical Scavenging Activity

The antioxidant activity was evaluated using the DPPH assay, following the method described by Brand-Williams et al. (1995) [49], with slight modifications. Briefly, 20 μL of each extract (200 μg/mL) was mixed with 180 μL DPPH solution (250 mM; ≥97%, Sigma-Aldrich, St. Louis, MO, USA) in a 96-well microplate (final volume 200 μL). The mixture was incubated at room temperature in the dark for 30 min, and absorbance was measured at 540 nm using a spectrophotometer (BioTek ELx800; BioTek Instruments Inc., Winnoski, VT, USA). Trolox (≥97%, Sigma-Aldrich, St. Louis, MO, USA) solutions were used as the references standard to construct a calibration curve (25–200 μM, y = −0.0009x − 0.0033 R^2^ = 0.982). The DPPH activity was expressed as mmol of Trolox equivalents per gram extract (mmol TE/g extract) and calculated according to the following Equation (4):

(4) $$\mathrm{DPPH}\;\mathrm{activity}\;\left(\frac{\mathrm{mmol}\;\mathrm{TE}}{g\;\mathrm{extract}}\right)=\frac{C_{\mathrm{TE}\;}\;\times \;\mathrm{DF}\;\times \;V}{C_{\mathrm{extract}}}$$

where *C* is the Trolox equivalent concentration obtained from the calibration curve (mmol/mL), *DF* is the dilution factor, *V* is the final volume of the reaction mixture (mL), and *C* is the concentration of the extract used in the assay (g/mL).

#### 4.7.2. ABTS^+^·Radical Scavenging Activity

The ABTS radical cation (ABTS^+)^ was generated by mixing 7 mM ABTS (≥98%, Sigma-Aldrich, St. Louis, MO, USA) with 2.45 mM potassium persulfate (≥99%, Sigma-Aldrich, St. Louis, MO, USA) in a 1:1 ratio and incubating in the dark at room temperature for 16 h [50]. Before use, the ABTS solution was adjusted to an appropriate absorbance. The antioxidant activity of the extracts (200 μg/mL) was determined in a 96-well microplate. Trolox solutions were used as the reference standard to construct a calibration curve (50–800 M, y = −0.0005x + 0.0312, R^2^ = 0.9865). The ABTS activity was expressed as mmol of Trolox equivalents per gram extract (mmol TE/g extract) and calculated according to the following Equation (5):

(5) $$\mathrm{ABTS}\;\mathrm{activity}\;\left(\frac{\mathrm{mg}\;\mathrm{TE}}{g\;\mathrm{extract}}\right)=\frac{C_{\mathrm{TE}\;}\;\times \;\mathrm{DF}\;\times \;V}{C_{\mathrm{extract}}}$$

where *C* is the Trolox equivalent concentration obtained from the calibration curve (mmol/mL), *DF* is the dilution factor, *V* is the final volume of the reaction mixture (mL), and *C* is the concentration of the extract used in the assay (g/mL).

#### 4.7.3. FRAP Ferric-Reducing Antioxidant Power Assay

The ferric-reducing antioxidant power was determined using the FRAP assay, as described by Benzie and Strain 1996 [51]. The FRAP reagent was freshly prepared by mixing 300 mM acetate buffer (pH 3.6; sodium acetate, ≥99%, Sigma-Aldrich, St. Louis, MO, USA; glacial acetic acid, ≥99.7%, Sigma-Aldrich, St. Louis, MO, USA), 10 mM TPTZ (≥99%, Sigma-Aldrich) in 40 mM HCl (37%, Sigma-Aldrich, St. Louis, MO, USA), and 20 mM FeCl_3_·6H_2_O (≥98%, Sigma-Aldrich, St. Louis, MO, USA) in a 10:1:1 ratio, and pre-warmed to 37 °C. In a 96-well microplate, 20 ul of extracts (200 μg/mL) or standard (FeSO_4_·7H_2_0) were added to 180 ul of FRAP reagent. The reaction mixture was incubated at 37 °C for 6 min, and absorbance was measured at 595 nm (BioTek ELx800; BioTek Instruments Inc., Winnoski, VT, USA). FeSO_4_·7H_2_0 solutions were used as the reference standard to construct a calibration curve (100–1000 M, y = −0.0002x + 0.0049, R^2^ = 0.9979). The FRAP activity was expressed as Fe^2+^ equivalents per gram of extract (mmol Fe^2+^/g extract) and calculated according to the following Equation (6):

(6) $$\mathrm{FRAP}\;\mathrm{activity}\;\left(\frac{\mathrm{mmol}\;{\mathrm{Fe}}^{2+}}{g\;\mathrm{extract}}\right)=\frac{C_{{\mathrm{Fe}}^{2+}\;}\;\times \;\mathrm{DF}\;\times \;V}{m}$$

where *C* is the Fe^2+^ equivalents concentration obtained from the calibration curve (mmol/mL), *DF* is the dilution factor, *V* is the final volume of the reaction mixture (mL), and *C* is the concentration of the extract used in the assay (g/mL).

### 4.8. Glucosidase Inhibition Assay

The α-glucosidase enzyme was isolated from the intestinal mucosa of adult male Wistar rats [52]. The intestinal tissue was obtained from the Animal House of the Academic Division of Health Sciences, UJAT. The mucosa was treated with 0.9% (*w*/*v*) saline solution (NaCl, ≥99.5%, Sigma-Aldrich, St. Louis, MO, USA), homogenized, and stored at 4 °C until use. All procedures were approved by the Institutional Review Board of the Academic Division of Health Sciences, UJAT (protocol number JI-PG095, June 2025).

In the enzymatic assay, the samples (extracts and fractions) were tested in triplicate at a concentration of 0.125 mg/mL, using 50% DMSO (Sigma-Aldrich, St. Louis, MO, USA) as the vehicle. Furthermore, acarbose (0.025 mg/mL; Sigma-Aldrich, St. Louis, MO, USA), *C. sinensis* extract (0.125 mg/mL), and 50% DMSO (negative control) were assessed. The reaction system (250 μL) consisted of: phosphate buffer (pH 7.0), a starch solution (8 μg/mL; Sigma-Aldrich, St. Louis, MO, USA), the sample, and 10 μL of the enzyme preparation. The reaction was initiated by adding the enzyme and incubating at 37 °C for 10 min. It was then terminated by adding 2 μL of acarbose and immediate cooling in an ice bath [52].

The released glucose was quantified using a microplate colorimetric method with a glucose oxidase/peroxidase kit (Thermo Fisher Scientific, Waltham, MA, USA). The plate was incubated at 37 °C for 30 min and subsequently read in a spectrophotometer (BioTek ELx800; BioTek Instruments Inc., Winnoski, VT, USA) at 505 nm. Enzyme activity was reported as the percentage of inhibition relative to the inhibitor-free control (100% enzymatic activity).

### 4.9. Pancreatic Lipase Inhibition Assay

The experimental reactions were prepared in triplicate for the *C. papaya* extracts or fractions (0.125 mg/mL), the vehicle (50% DMSO), *C. sinensis* extract (0.125 mg/mL), and orlistat (1 μg/mL; Sigma-Aldrich, St. Louis, MO, USA). These concentrations are based on the methodology of a previous study [52].

The assay consisted of a colorimetric reaction in a microplate incubated at 37 °C. The following reaction system was used: 0.2 mM DMPTB (282413, Sigma-Aldrich, St. Louis, MO, USA), 0.8 mM DTNB (D-8130, Sigma-Aldrich, St. Louis, MO, USA), 0.1 M NaCl (≥99.5%, Sigma-Aldrich), 2 mM CaCl_2_ (≥99%, Sigma-Aldrich), 0.04% Triton X-100 (X100, Sigma-Aldrich, St. Louis, MO, USA), and 65 μg/mL type II porcine pancreatic lipase (L3126, Sigma-Aldrich, St. Louis, MO, USA) in Tris-HCl buffer (pH 7.0), with a final volume of 1 mL [53]. The enzymatic kinetics were recorded using a spectrophotometer (BioTek ELx800; BioTek Instruments Inc., Winnoski, VT, USA) by measuring absorbance at 412 nm every 20 s. Finally, the initial reaction velocity (V_0_) was determined from the slope of the absorbance versus time curve, in accordance with Michaelis–Menten kinetic principles [54].

### 4.10. Computational ADMET Analysis

The ADMETlab 3.0 web server (https://admetlab3.scbdd.com/, accessed 29 June 2026) was used to estimate the pharmacokinetic profiles and physicochemical properties of clitorin and loliolide. Canonical SMILES (Simplified Molecular-Input Line-Entry System) codes for these compounds were obtained from the PubChem database.

### 4.11. Statistical Analyses

Comparisons between multiple treated groups and a single control group were performed using Dunnett’s post hoc test. In all cases, differences were considered statistically significant at *p* ≤ 0.05. Enzyme inhibition percentages were plotted against the logarithm of concentration using GraphPad Prism version 10 (GraphPad Software LLC, Boston, MA, USA), and IC_50_ values were calculated by fitting the data to a nonlinear sigmoidal curve. Additionally, the type of inhibition was assessed by plotting the initial velocities in Lineweaver–Burk coordinates (1/V vs. 1/[S]).

## Figures and Tables

**Table 1:** Total compound content of *C. papaya* leaf extracts.

Chemical Content	CPHX	CPDM	CPME	CPHA
Total phenolic content (mg GAE per g of extract)	11.42 ± 1.66 ^c^	47.05 ± 2.60 ^b^	254.10 ± 6.83 ^a^	239.70 ± 4.61 ^a^
Total flavonoid content (mg RE per g of extract)	-	104.11 ± 14.0 ^a^	139.41 ± 38.87 ^ab^	175.66 ± 23.41 ^b^
Total terpene content (mg ME per g of extract)	51.47 ± 6.47 ^a^	39.08 ± 7.27 ^b^	10.39 ± 2.70 ^c^	2.34 ± 0.56 ^d^

Values expressed are means ± S.D of three parallel measurements; ^a–d^ means in the same row not sharing the same superscript are significantly different *(p* ≤ 0.05); GAE: gallic acid equivalent; RE: rutin equivalent.

**Table 2:** Antioxidant activity of *C. papaya* extracts.

Assay	CPHA	CPME	CPDM	CPHX
DPPH (mmol TE per g of extract)	5.99 ± 0.49 ^b^	8.99 ± 0.51 ^a^	6.55 ± 0.57 ^b^	6.40 ± 0.33 ^b^
ABTS^+^· (mmol TE per g of extract)	34.47 ± 3.66 ^a^	35.94 ± 0.28 ^a^	29.94 ± 1.43 ^b^	21.72 ± 3.65 ^c^
FRAP (mmol Fe^2+^ per g of extract)	6.34 ± 1.56 ^d^	48.62 ± 1.72 ^a^	22.43 ± 7.11 ^c^	37.81 ± 2.72 ^b^

Values expressed are means ± S.D. of three parallel measurements; ^a–d^ means in the same row not sharing the same superscript are significantly different (*p* ≤ 0.05); TE: trolox equivalent; Fe^2+^: Fe(II) equivalent.

**Table 3:** In vitro α-glucosidase inhibitory activity of *C. papaya* leaf extracts.

Group	Sample	% of Inhibition	vs. Acarbose	vs. *C. sinensis*
Extracts (0.125 mg/mL)	CPHX	24.54 ± 3.87	*p* < 0.001	*p* < 0.001
	CPDM	2.47 ± 2.82	*p* < 0.001	*p* < 0.001
	CPME	12.8 ± 0.82	*p* < 0.001	*p* < 0.001
	CPHA	38.44 ± 5.94	*p* = 0.002	*p* < 0.001
Controls	Acarbose	47.82 ± 1.67	-	*p* < 0.001
	*C. sinensis*	57.32 ± 2.86	*p* < 0.001	-

CPHX, hexane extract; CPDM, dichloromethane extract; CPME, methanol extract; CPHA, hydroalcoholic extract.

**Table 4:** In vitro α-glucosidase inhibitory activity of CPHA fractions.

Group	Sample	% of Inhibition	vs. Acarbose	vs. *C. sinensis*
Fractions of CPHA (0.125 mg/mL)	CpC7-R1	32.74 ± 6.87	*p* = 0.010	*p* < 0.001
	CpC7-R2	30.83 ± 7.35	*p* = 0.025	*p* < 0.001
	CPC7-R3	27.88 ± 2.65	*p* < 0.001	*p* < 0.001
	CpC7-R4	5.27 ± 5.23	*p* < 0.001	*p* < 0.001
Fractions of CPC7-R1 (0.125 mg/mL)	CPC8-R2	27.11 ± 1.56	*p* < 0.001	*p* < 0.001
	CpC8-R6	31.61 ± 4.10	*p* = 0.015	*p* < 0.001
	CpC8-R7	30.11 ± 1.72	*p* = 0.030	*p* < 0.001
	CpC8-R10	34.37 ± 3.11	*p* = 0.008	*p* < 0.001
	CpC8-R11	28.86 ± 5.21	*p* = 0.002	*p* < 0.001
Fractions of CPC8-R10 (0.125 mg/mL)	CPC10-R3	27.93 ± 5.90	*p* < 0.001	*p* < 0.001
	CPC10-R7	7.26 ± 4.65	*p* < 0.001	*p* < 0.001
	CPC10-R10	30.32 ± 4.85	*p* = 0.002	*p* < 0.001
	CPC10-R12	32.72 ± 3.81	*p* = 0.001	*p* < 0.001
Fractions of CPC10-R12 (0.125 mg/mL)	CPC11-R4	28.92 ± 7.84	*p* = 0.003	*p* < 0.001
	CPC11-R10	25.40 ± 2.99	*p* < 0.001	*p* < 0.001
	CPC11-R12	47.23 ± 9.62	*p* = 0.999	*p* = 0.035
	CPC11-R15	39.82 ± 3.03	*p* = 0.045	*p* < 0.001
Controls	Acarbose (0.025 mg/mL)	47.82 ± 1.67	-	*p* < 0.001
	*C. sinensis* (0.125 mg/mL)	57.32 ± 2.86	*p* < 0.001	-

**Table 5:** Phytochemical profile of the hydroalcoholic extract and fractions.

Sample	*t*_R_	λ_max_	Compound
CPC7-R1	9.05	215.4, 255.6, 353.4	Myricetin tetraglycoside
CPHA	9.42	203.7, 256.8, 352.2	Quercetin glycoside
CPC7-R1,CPC10-R12	9.48	215, 265.1, 346.2	Kaempferol glycoside
CPC11-R12	9.53	210.7, 265.1, 347.4	Clitorin
CPC8-R10	9.53	209.6, 265.1, 346.2	Kaempferol glycoside
CPHA	9.58	196.6, 237.9, 329.5	Caffeic acid
CPC7-R1, CPC8-R10, CPC10-R12	9.80	213.1, 255.6, 354.6	Rutin
CPHA	9.90	199.0, 255.1, 348.6	Kaempferol glycoside
CPC7-R1	10.03	206.0, 237.9, 328.3	Ferulic acid
CPHA	10.33	208.4, 255.6, 352.2	Quercetin glycoside
CPC7-R1	10.47	265.1, 300.8, 348.6	Kaempferitrin
CPC8-R10, CPC10-R12	10.91	193.1, 227.2, 323.5	Caffeic acid derivative
CPHA	10.97	194.3, 239.1, 325.9	Caffeic acid derivative
CPC7-R1,CPC10-R12	12.80	215.4, 266.3, 313.9	Tiliroside
CPHA	13.95	219.0, 343.8	Coumarin
CPHA	25.65	220.2, 335.5	Coumarin

CPHA, hydroalcoholic extract.

**Table 6:** Inhibitory activity of extracts and fractions from the most active extract on pancreatic lipases.

Groups	Samples	% of Inhibition	vs. Orlistat	vs. *C. sinensis*
Extracts (0.125 mg/mL)	CPHX	12.59 ± 6.61	*p* < 0.001	*p* < 0.001
	CPDM	52.2 ± 9.74	*p* = 0.999	*p* = 0.999
	CPME	30.77 ± 9.42	*p* < 0.001	*p* < 0.001
	CPHA	1.89 ± 2.67	*p* < 0.001	*p* < 0.001
Fractions of CPDM (0.125 mg/mL)	CPC1-R2	83.33 ± 4.28	<0.001	<0.001
	CPC1-R4	11.37 ± 5.56	<0.001	<0.001
	CPC1-R7	1.51 ± 2.14	<0.001	<0.001
	CPC1-R9	75.52 ± 5.96	<0.001	<0.001
	CPC1-R11	78.03 ± 9.62	<0.001	<0.001
Fractions of CPC1-R11 (0.125 mg/mL)	CPC6-R2	20.51 ± 7.80	*p* < 0.001	*p* < 0.001
	CPC6-R345	20.65 ± 9.34	*p* < 0.001	*p* < 0.001
	CPC6-R9	94.23 ± 5.00	*p* < 0.001	*p* < 0.001
	CPC6-R12	34.31 ± 5.71	*p* = 0.015	*p* < 0.001
	CPC6-R15	49.75 ± 8.23	*p* = 0.999	*p* = 0.5
Controls	Orlistat (1 µg/mL)	48.03 ± 7.12	-	*p* = 0.5
	*C. sinensis* (0.125 mg/mL)	53.51 ± 6.24	*p* = 0.5	-

CPHX, hexane extract; CPDM, dichloromethane extract; CPME, methanol extract; CPHA, hydroalcoholic extract.

**Table 7:** Compounds identified by CG-MS of CPDM and the most active fraction.

Sample	*t_R_* (min)	Peak Area (%)	Name of Compounds	MF	Structure of Compounds
CPC6-R9	15.79	100	6-Hydroxy-4,4,7a-trimethyl-5,6,7,7a-tetrahydro-1-benzofuran-2(4H)-one	854	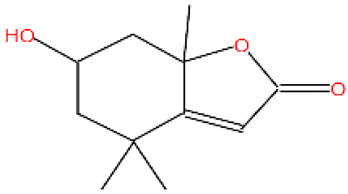
CPDM	17.07	55.33	Cyclopropanebutanoic acid, 2-[[2-[[2-[(2-pentylcyclopropyl)methyl]cyclopropyl]methyl]cyclopropyl]methyl]-, methyl ester	719	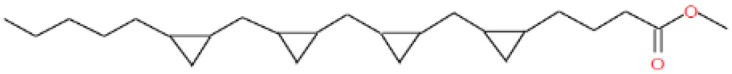
CPDM	18.70	44.67	Benz[e]azulene-3,8-dione, 3a,4,6a,7,9,10,10a,10b-octahydro-3a,10a-dihydroxy-5-(hydroxymethyl)-7-(1-hydroxy-1-methylethyl)-2,10-dimethyl-	668	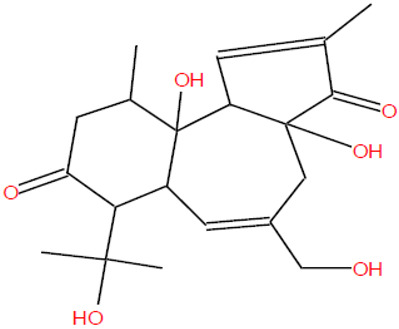
CPDM	18.70	44.67	2-Bromotetradecanoic acid	653	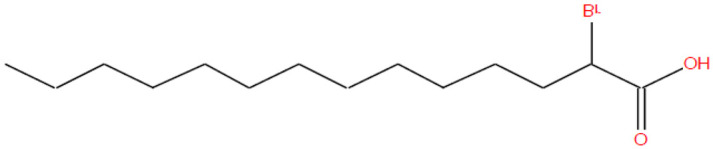

CPDM, dichloromethane extract; *t_R_*, retention time; MF, match factor.

**Table 8:** Predicted pharmacokinetic properties profile of clitorin and loliolide.

Compound	Physicochemical Properties	Absorption	Distribution	Metabolism	Excretion and Toxicity
Name: Clitorin Formula: C_33_H_40_O_19_	Molecular weight: 740.22 Da TPSA: 308.12 Å^2^ HBA: 19 HBD: 11 Rotatable bonds: 8 logS: −2.42	Human intestinal absorption: Negative Caco-2 permeability: Low	Plasma protein binding: 82.0% BBB penetration: Low probability	CYP2C8: Predicted inhibitor	Plasma clearance: 0.98 mL/min/kg hERG Blockers: 0.01
Name: Loliolide Formula: C_11_H_16_O_3_	Molecular weight: 196.11 Da logP: 1.35 TPSA: 46.53 Å^2^ HBA: 3 HBD: 1 Rotatable bonds: 0 logS: −2.89	Human intestinal absorption: Positive Caco-2 permeability: Acceptable	Plasma protein binding: 73.3% BBB penetration: Low probability	CYP3A4: Predicted substrate/inhibitor CYP2C19: Predicted substrate	Plasma clearance: 7.40 mL/min/kg Blockers: 0.05

HBA, hydrogen bond acceptors; HBD, hydrogen bond donors; TPSA, topological polar surface area; logP, octanol/water partition coefficient; logS, aqueous solubility; Caco-2, human colorectal adenocarcinoma cell permeability model; BBB, blood–brain barrier; CYP, cytochrome P450.

## Data Availability

The original contributions presented in this study are included in the article. Further inquiries can be directed to the corresponding author.

## Supplementary Materials

The following supporting information can be downloaded at: https://www.mdpi.com/article/10.3390/molecules31132394/s1.

## Abbreviations

The following abbreviations are used in this manuscript:

ABTS	2,2′-Azino-bis(3-ethylbenzothiazoline-6-sulfonic acid)
CPHA	Hydroalcoholic extract of *Carica papaya* leaves
CPDM	Dichloromethane extract of *Carica papaya* leaves
CPME	Methanol extract of *Carica papaya* leaves
CPHX	Hexane extract of *Carica papaya* leaves
DPPH	2,2-Diphenyl-1-picrylhydrazyl
FRAP	Ferric-Reducing Antioxidant Power
GAE	Gallic Acid Equivalent
GC-MS	Gas Chromatography–Mass Spectrometry
HPLC-PDA	High-Performance Liquid Chromatography coupled with Photodiode Array Detection
IC_50_	Half Maximal Inhibitory Concentration
RE	Rutin Equivalent
TE	Trolox Equivalent
*t_R_*	Retention time
UPLC-MS/MS	Ultra-Performance Liquid Chromatography–Mass Spectrometry
