# Supplementary material for: Phytochemical Analysis, Antioxidant Activity, and Inhibition of Digestive Enzymes of Carica papaya L. Leaf
Source: Molecules. 2026 Jul 7;31(13):2394. doi: 10.3390/molecules31132394 (PMC13363069; doi:10.3390/molecules31132394)
Supplement: Supplementary file 1 [file molecules-31-02394-s001.zip › molecules-4391788-supplementary.pdf]

Supplementary Material

Phytochemical Analysis, Antioxidant Activity, and Inhibition of Digestive Enzymes of *Carica papaya* L. Leaf

1. Phytochemical analysis of the hydroalcoholic extract of *C. papaya*

Figures S1 to S5 present the chromatograms (monitored at 340 nm) and corresponding UV-Vis absorption spectra from the bioassay-guided HPLC-UV-Vis fractionation of the hydroalcoholic extract of *C. papaya* leaves. To confirm the chromatographic purity of the isolated bioactive compound, additional HPLC-UV-Vis analyses were performed at 345 nm (Figure S6). The reference standards used for peak/compound identification are shown in Figures S7–S11.

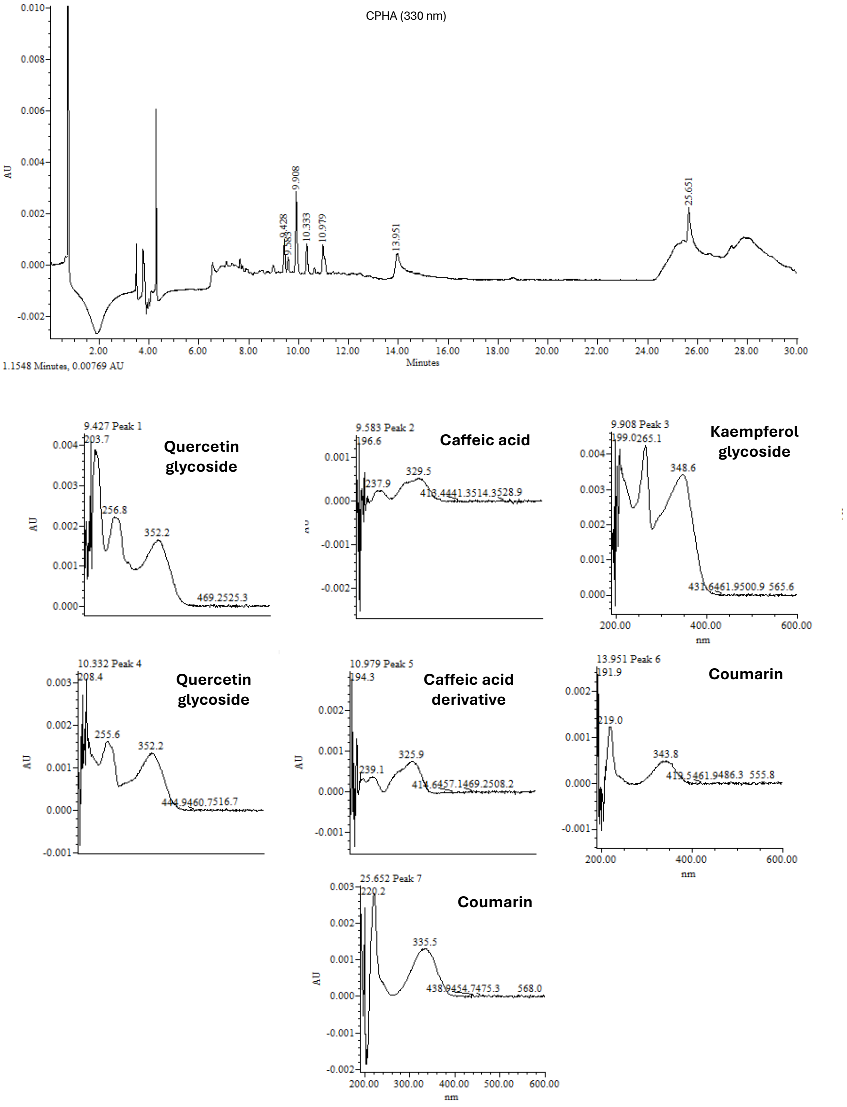

Figure S1. Chromatogram and UV–Vis absorption spectra of the hydroalcoholic extract of *C. papaya* leaves.

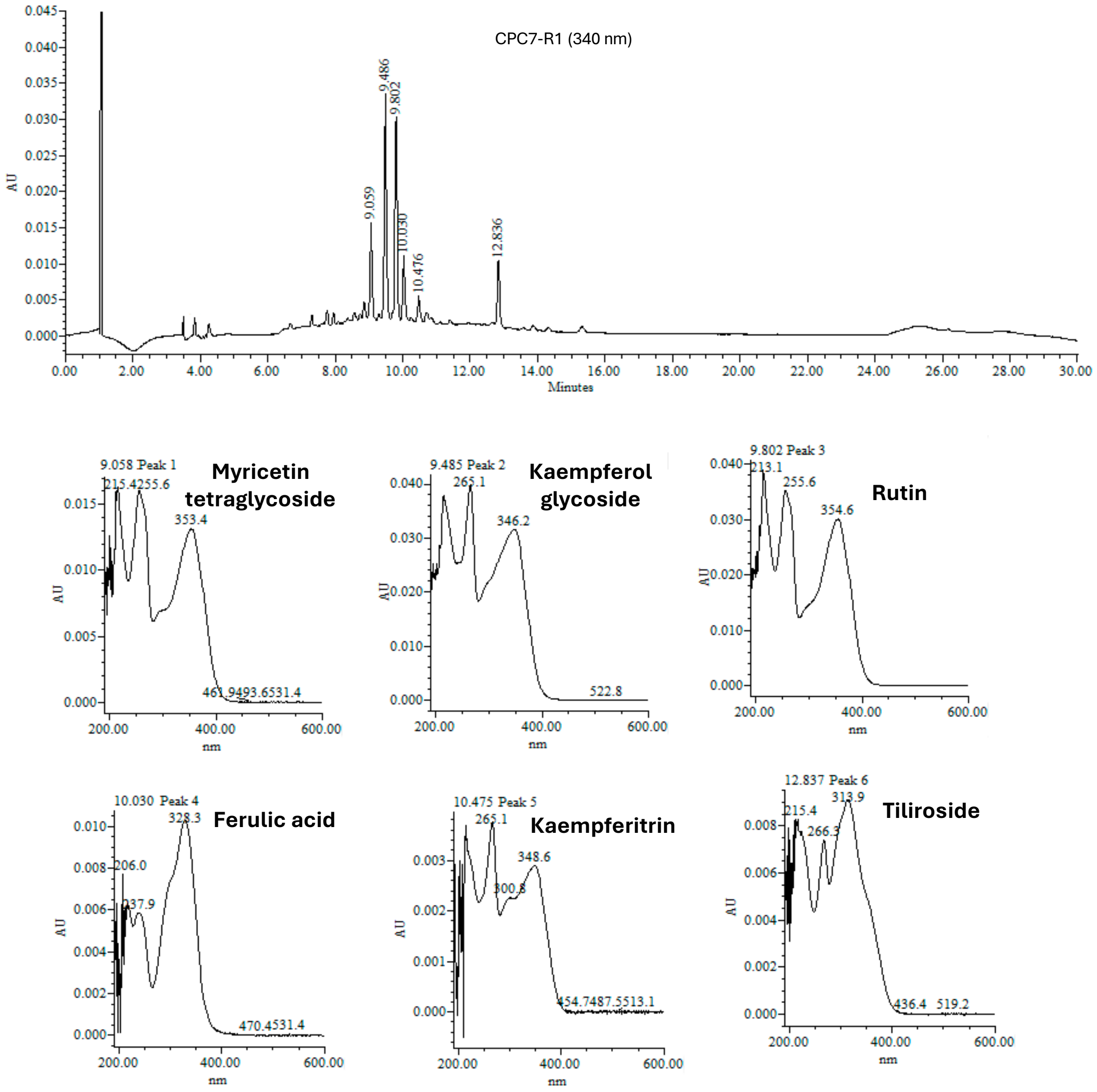

Figure S2. Chromatogram and UV–Vis absorption spectra of the CPC7-R1 fraction derived from the hydroalcoholic extract of *C. papaya* leaves.

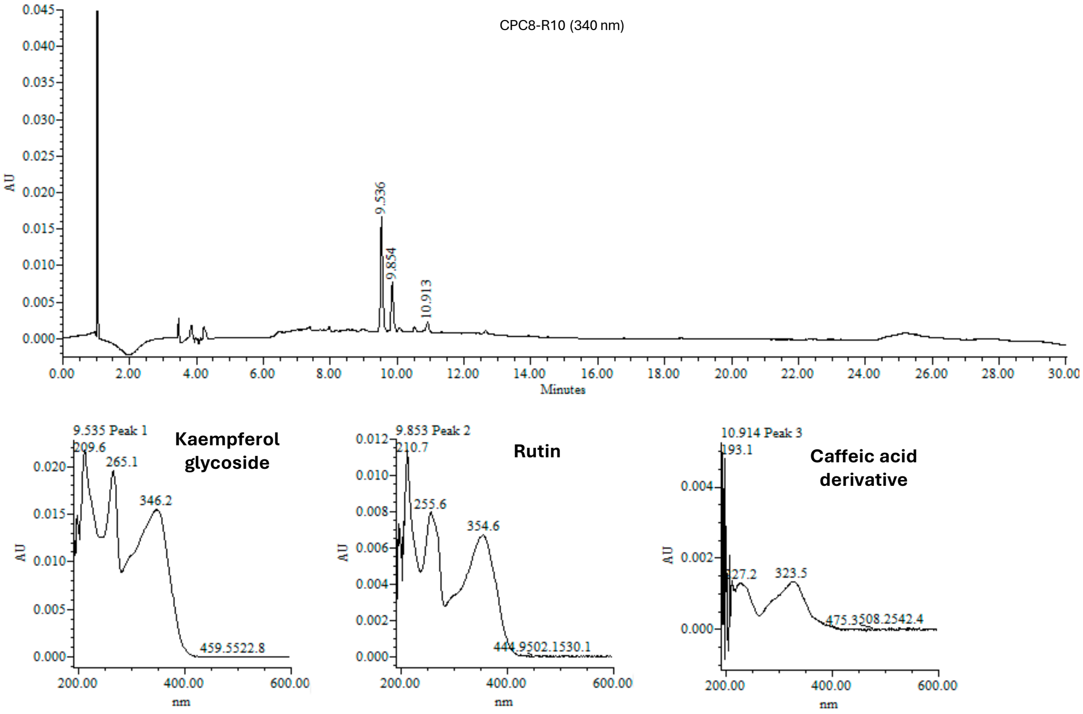

Figure S3. Chromatogram and UV–Vis absorption spectra of the CPC8-R10 fraction derived from the hydroalcoholic extract of *C. papaya* leaves.

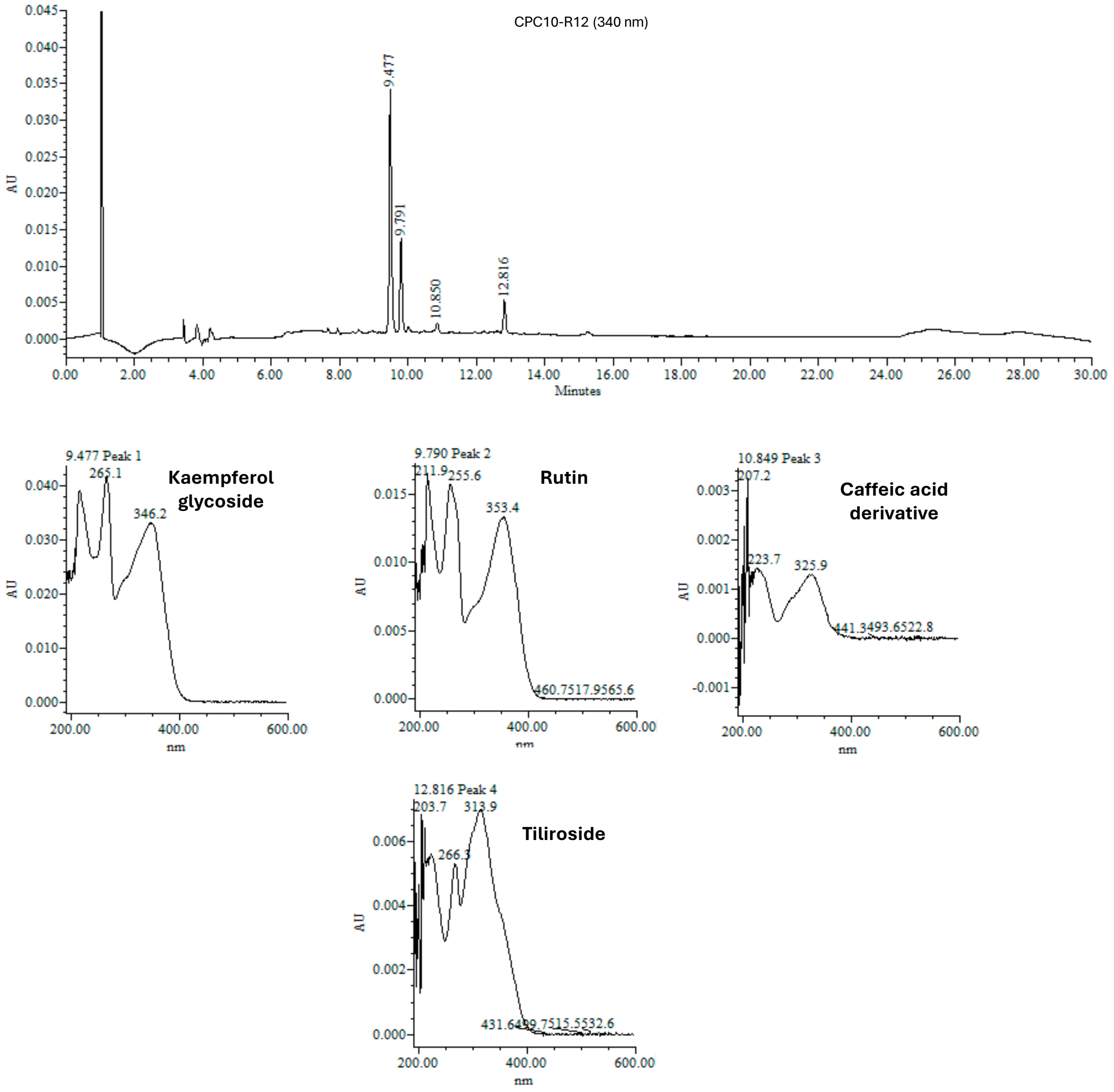

Figure S4. Chromatogram and UV–Vis absorption spectra of the CPC10-R12 fraction derived from the hydroalcoholic extract of *C. papaya* leaves.

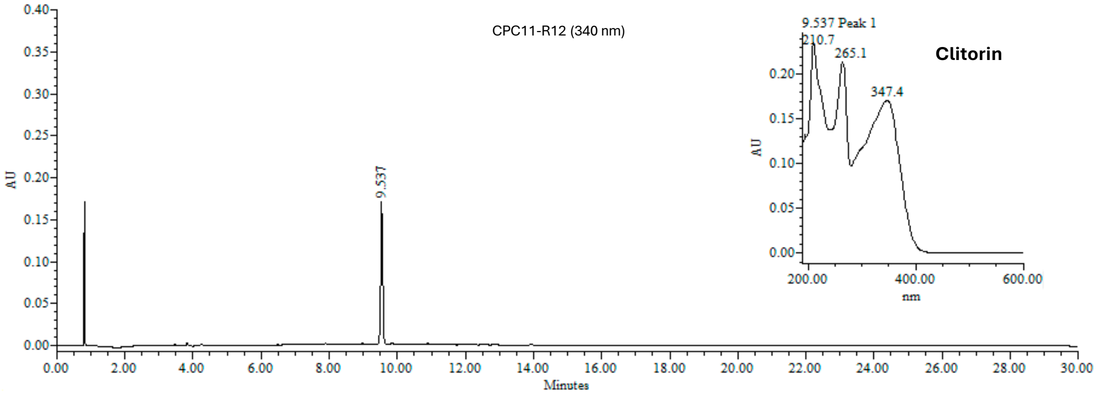

Figure S5. Chromatogram and UV–Vis absorption spectra of the CPC11-R12 fraction derived from the hydroalcoholic extract of *C. papaya* leaves. The peak was initially detected as a kaempferol derivative and was subsequently characterized as clitorin.

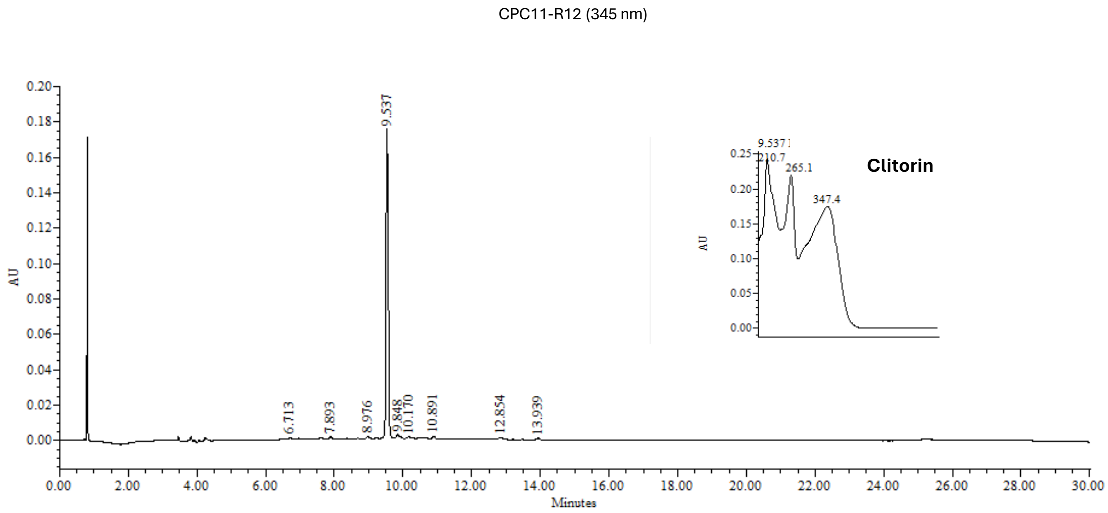

Figure S6. HPLC-UV-Vis chromatogram and UV-Vis absorption spectrum of the purified bioactive compound (fraction CPC11-R12), monitored at 345 nm.

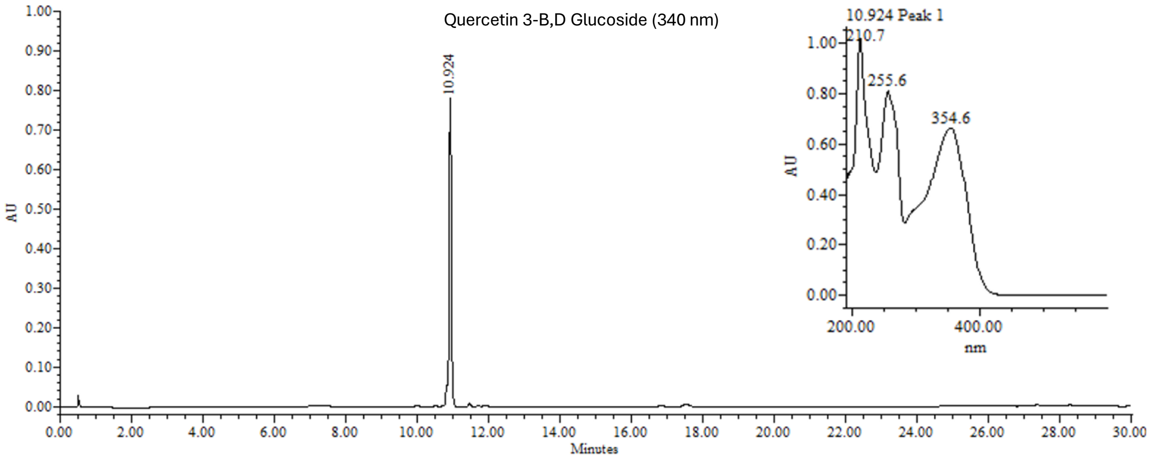

Figure S7. Chromatogram and UV–Vis absorption spectra of the quercetin 3-β-D-glucoside standard.

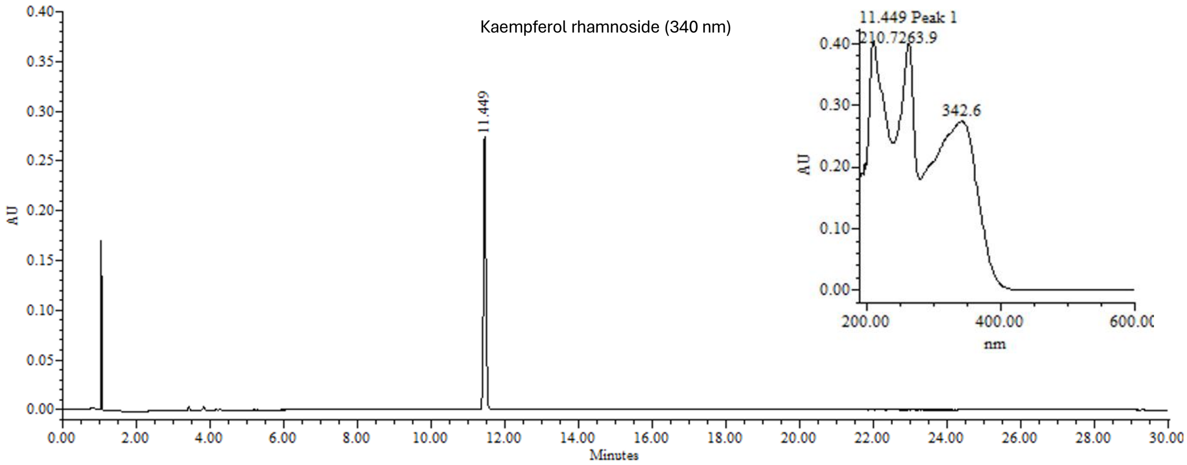

Figure S8. Chromatogram and UV–Vis absorption spectra of the kaempferol rhamnoside standard.

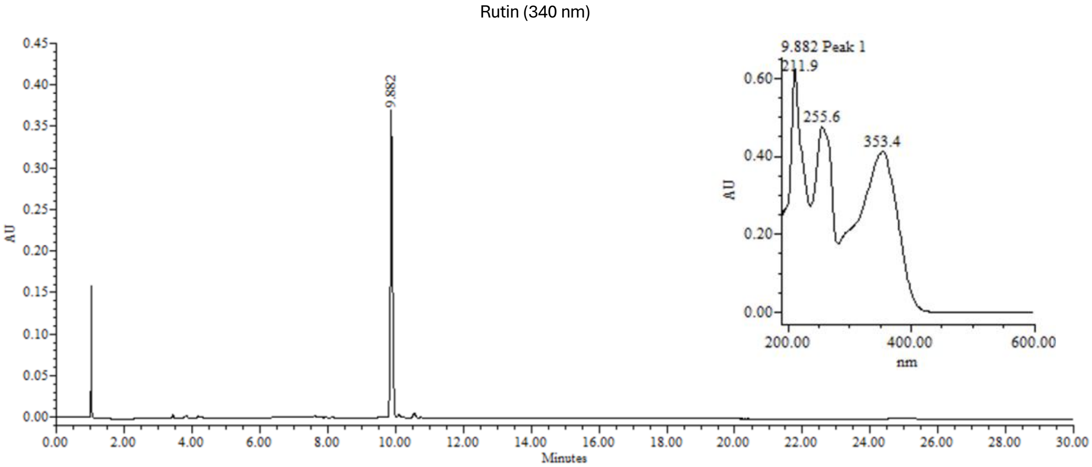

Figure S9. Chromatogram and UV–Vis absorption spectra of the rutin standard.

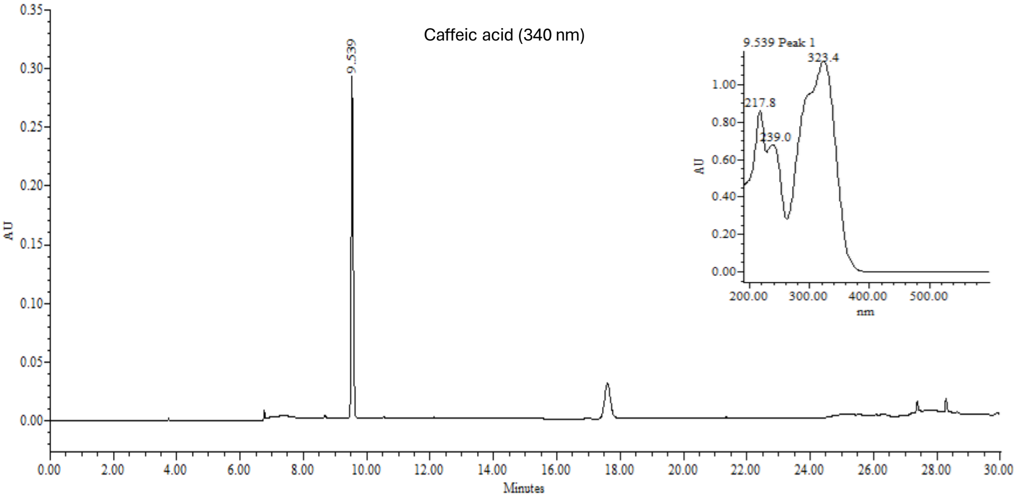

Figure S10. Chromatogram and UV–Vis absorption spectra of the caffeic acid standard.

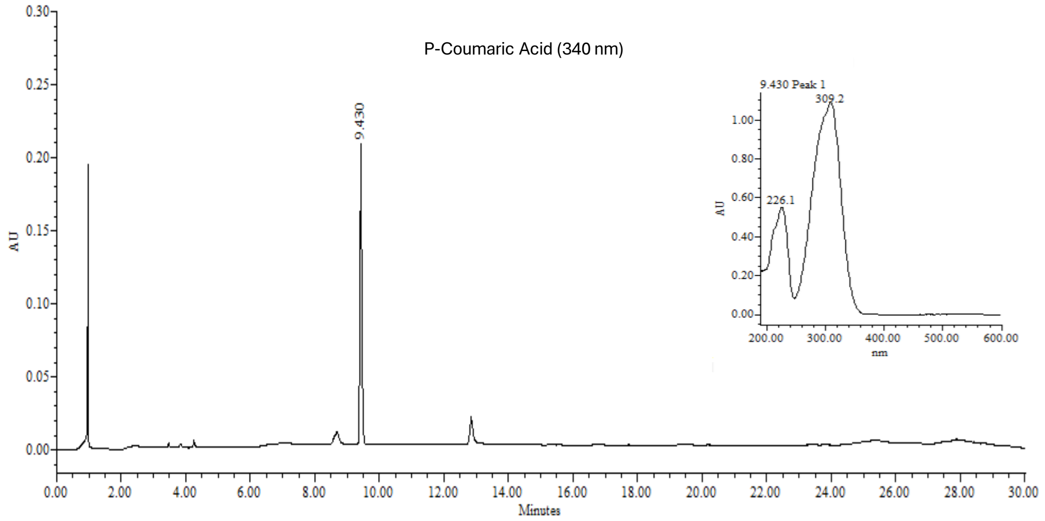

Figure S11. Chromatogram and UV–Vis absorption spectra of the p-coumaric acid standard.

2. Bioactive compounds present in the diclorometHane extract of *C. papaya*

The dichloromethane extract of *C. papaya* was analyzed by GC-MS. The resulting chromatogram is shown in Figure S12, and the mass spectra of the observed peaks are presented in Figures S13–S15. Notably, for peak 2, the analysis showed correlation factors of 668 for the compound benz[e]azulene-3,8-dione, 3a,4,6a,7,9,10,10a,10b-octahydro-3a,10a-dihydroxy-5-(hydroxymethyl)-7-(1-hydroxy-1-methylethyl)-2,10-dimethyl- and 653 for the compound 2-bromotetradecanoic acid. Additionally, the chromatogram of the most active fraction and the mass spectrum of the corresponding peak are shown in Figures S16 and S17, respectively.

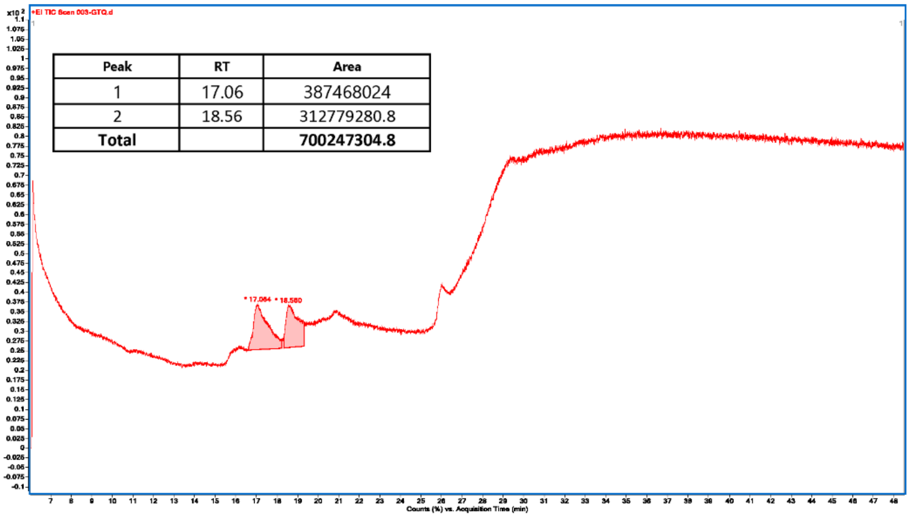

Peak	RT	Area
1	17.06	387468024
2	18.56	312779280.8
**Total**		**700247304.8**

Figure S12. GC-MS chromatogram of the dichloromethane extract of *Carica papaya* leaves, showing different compounds at various retention times.

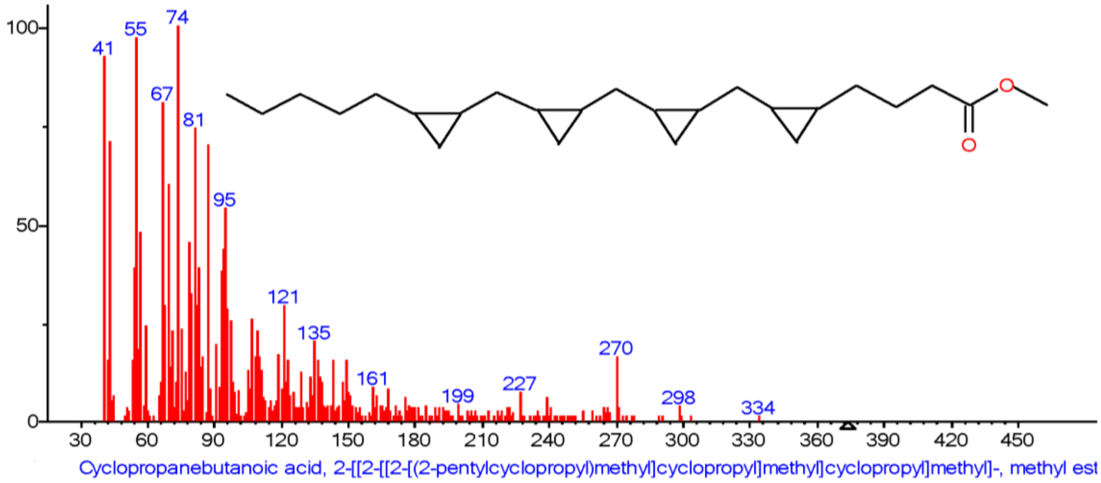

Figure S13. Mass spectrum of peak 1 observed in the dichloromethane extract of *C. papaya*.

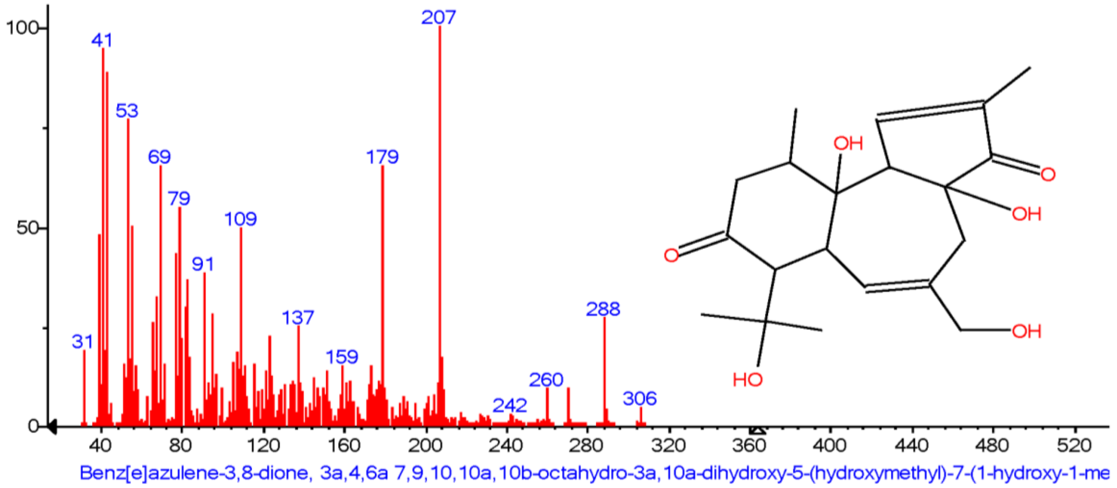

Figure S14. Mass spectrum of peak 2a (benz[e]azulene-3,8-dione derivative; correlation factor 668) observed in the dichloromethane extract of *C. papaya*.

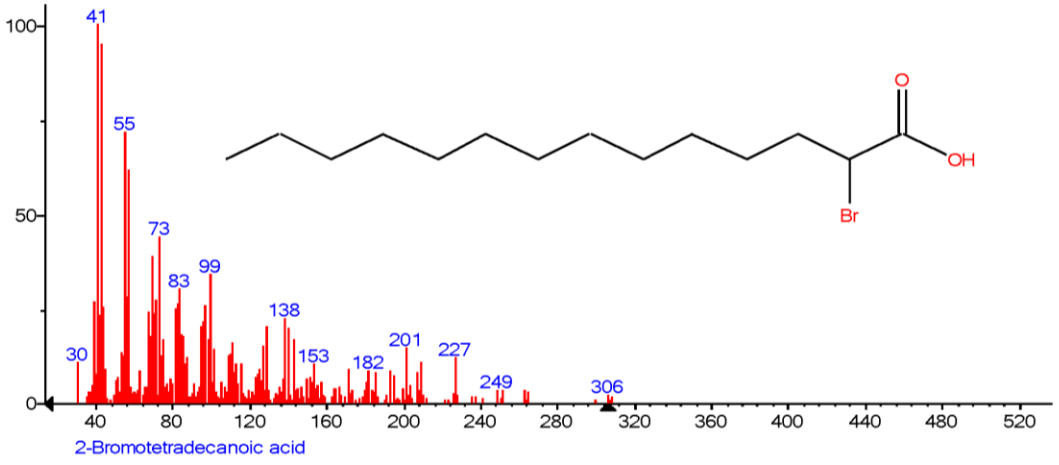

Figure S15. Mass spectrum of peak 2b (2-bromotetradecanoic acid; correlation factor 653) observed in the dichloromethane extract of *C. papaya*.

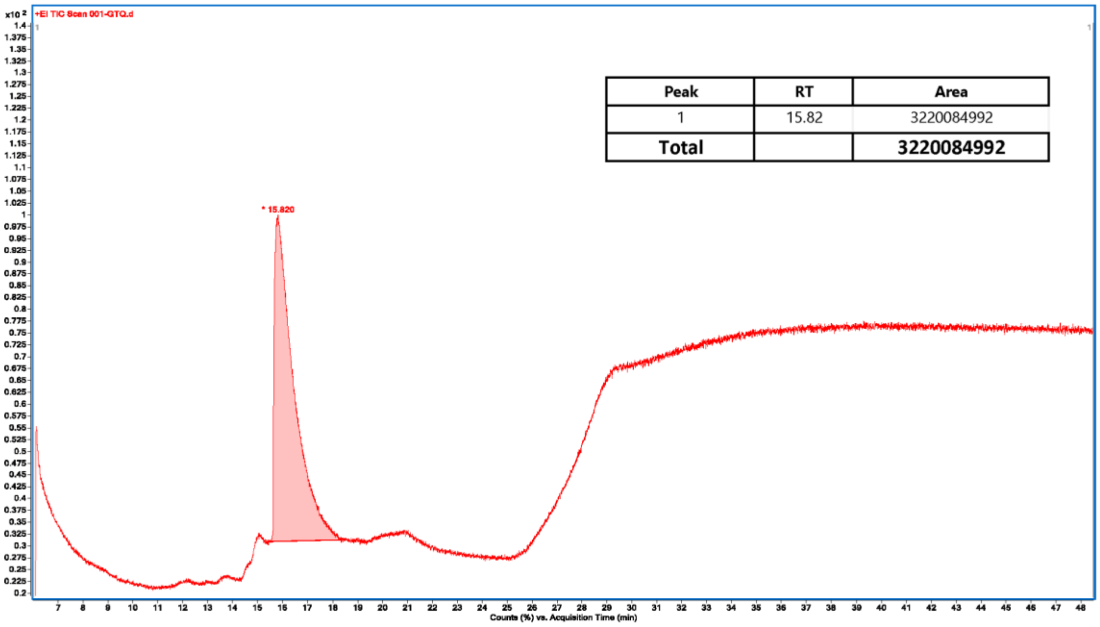

Figure S16. GC-MS chromatogram of the most active fraction from the dichloromethane extract of *C. papaya*.

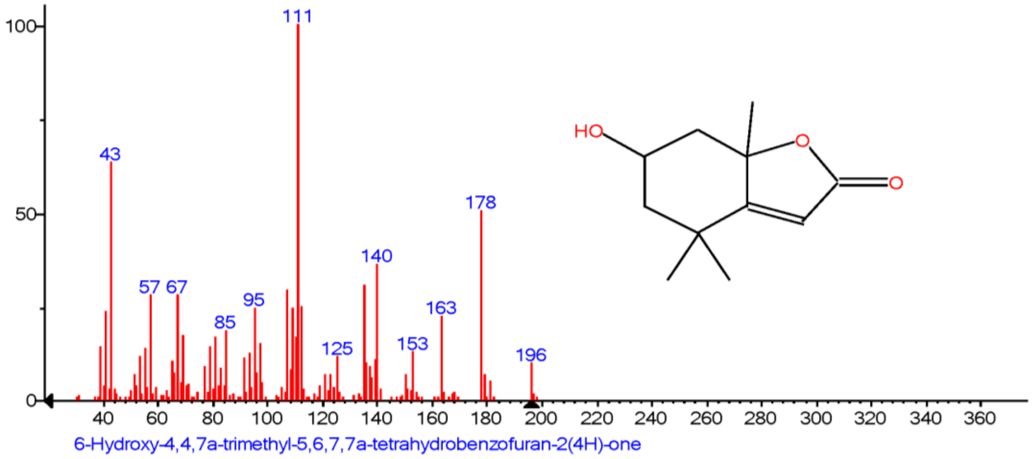

Figure S17. Mass spectrum of the peak observed in the most active fraction.

3. NMR Spectra of Loliolide

Figures S18 and S19 show the ^{1}H and ^{13}C MNR spectra of loliolide acquired in $CDCl_3$, which were used to confirm the identity of the isolated compound.

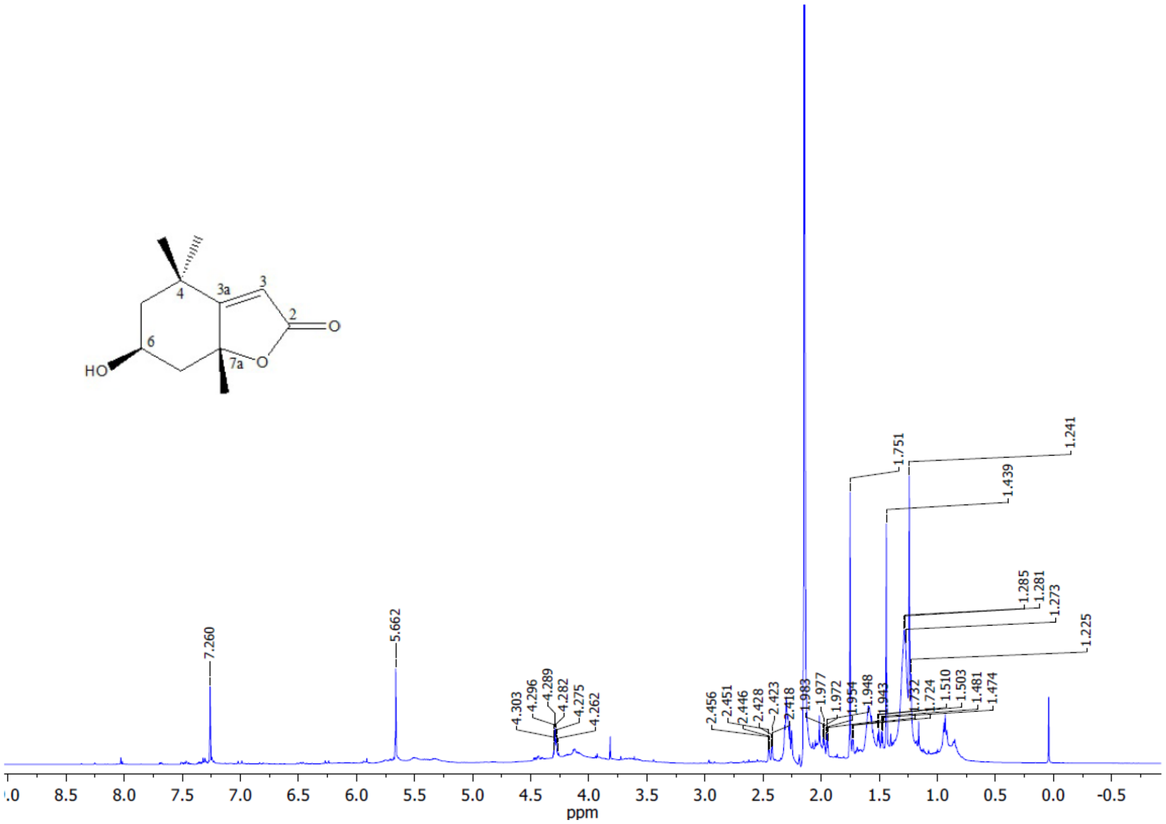

Figure S18. ^{1}H-NMR (CDCl3, 500 MHz) of Loliolide.

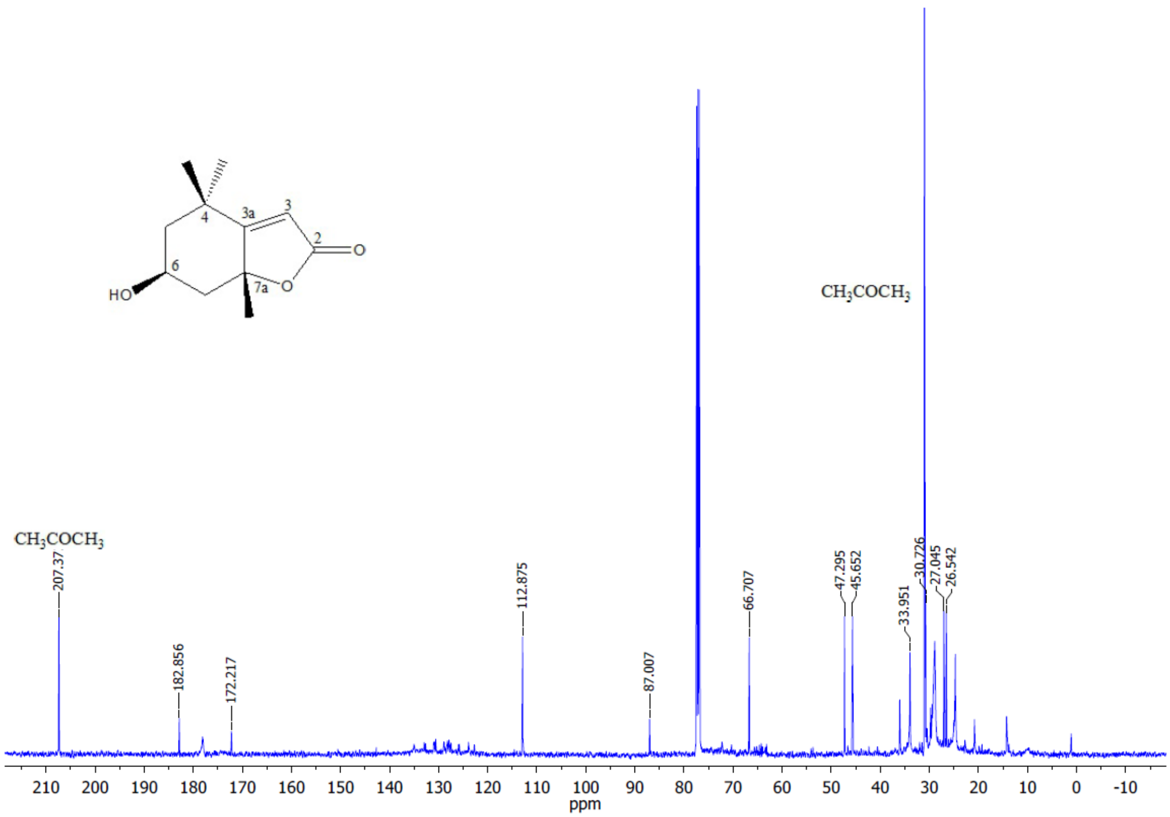

Figure S19. ^{13}C-NMR (CDCl3, 125 MHz) of Loliolide.

4. ADMET evaluation of loliolide

The SMILES string of loliolide (CC1(C)CC@HC[C@@]2(C)OC(=O)C=C12) was submitted to ADMETlab 3.0 for ADMET prediction. The complete prediction results are presented in Tables S1–S6. The color-coded decision indicators used throughout the tables are interpreted as follows: green, excellent; amber, medium; and red, poor.

Table S1. Physicochemical Property.

Property	Value	Comment
Molecular Weight	196.11	Contain hydrogen atoms. Optimal:100~600
Volume	202.797	Van der Waals volume
Density	0.967	Density = MW / Volume
nHA	3.0	Number of hydrogen bond acceptors. Optimal:0~12
nHD	1.0	Number of hydrogen bond donors. Optimal:0~7
nRot	0.0	Number of rotatable bonds. Optimal:0~11
nRing	2.0	Number of rings. Optimal:0~6
MaxRing	9.0	Number of atoms in the biggest ring. Optimal:0~18
nHet	3.0	Number of heteroatoms. Optimal:1~15
fChar	0.0	Formal charge. Optimal:-4 ~4
nRig	11.0	Number of rigid bonds. Optimal:0~30
Flexibility	0.0	Flexibility = nRot /nRig
Stereo Centers	2.0	Stereo Centers. Optimal: □ 2
TPSA	46.53	Topological Polar Surface Area. Optimal:0~140
logS	-2.893	The logarithm of aqueous solubility value.
logP	1.348	The logarithm of the n-octanol/water distribution coefficients at pH=7.4.
logD	1.371	The logarithm of the n-octanol/water distribution coefficient.
pka (Acid)	8.193	Acid-base dissociation constant (pKa) value represents the strength of a drug molecule's acidity or basicity.
pka (Base)	3.117	Acid-base dissociation constant (pKa) value represents the strength of a drug molecule's acidity or basicity.
Melting point	199.073	The predicted melting point of a compound is expressed in degrees Celsius (°C).<br>Melting points below 25°C are classified as liquids, while melting points above 25°C are classified as solids.
Boiling point	309.741	The predicted melting point of a compound is expressed in degrees Celsius (°C).<br>A normal boiling point below 25°C is categorized as a gas.

Table S2. Absorption.

Property	Value	Decision	Comment
Caco-2 Permeability	-4.926	🟢	Optimal: higher than -5.15 Log unit
MDCK Permeability	-4.76	🔴	■ low permeability: < 2 × 10-6 cm/s<br>■ medium permeability: 2-20 × 10^{-6} cm/s<br>■ high passive permeability: > 20 × 10^{-6} cm/s
PAMPA	0.945	🔴	■ The experimental data for Peff was logarithmically transformed (logPeff).<br>■ Molecules with log Peff values below 2.0 were classified as low-permeability (Category 0), while those with log Peff values exceeding 2.5 were classified as high-permeability (Category 1).
Pgp-inhibitor	0.368	🟠	■ Category 1: Inhibitor;<br>■ Category 0: Non-inhibitor;<br>■ The output value is the probability of being Pgp-inhibitor
Pgp-substrate	0.284	🟢	■ Category 1: substrate;<br>■ Category 0: Non-substrate;<br>■ The output value is the probability of being Pgp-substrate
HIA	0.377	🟠	■ Human Intestinal Absorption<br>■ Category 1: HIA+(HIA < 30%);<br>■ Category 0: HIA-(HIA >= 30%);<br>■ The output value is the probability of being HIA+
$F_{20\%}$	0.958	🔴	■ 20% Bioavailability<br>■ Category 1: F 20% + (bioavailability < 20%);<br>■ Category 0: F 20% - (bioavailability □ 20%);<br>■ The output value is the probability of being F 20% +
$F_{30\%}$	0.876	🔴	■ 30% Bioavailability<br>■ Category 1: F 30% + (bioavailability < 30%);<br>■ Category 0: F 30% - (bioavailability □ 30%);<br>■ The output value is the probability of being F 30% +
$F_{50\%}$	0.977	🔴	■ 50% Bioavailability<br>■ Category 1: F 50% + (bioavailability < 50%);<br>■ Category 0: F 50% - (bioavailability □ 50%);<br>■ The output value is the probability of being F 50% +

Table S3. Distribution.

Property	Value	Decision	Comment
PPB	73.277	🟢	■ Plasma Protein Binding<br>Optimal: < 90%.<br>■ Drugs with high protein-bound may have a low therapeutic index.
VDss	-0.117	🔴	■ Volume Distribution<br>■ Optimal: 0.04-20L/kg
BBB	0.19	🟢	■ Blood-Brain Barrier Penetration<br>■ Category 1: BBB+; Category 0: BBB-;<br>■ The output value is the probability of being BBB+
Fu	25.103	🟢	■ The fraction unbound in plasms<br>■ Low: <5%; Middle: 5~20%; High: > 20%
OATP1B1 inhibitor	0.971	🔴	■ Category 0: Non-inhibitor; Category 1: inhibitor.<br>■ The output value is the probability of being inhibitor, within the range of 0 to 1.
OATP1B3 inhibitor	0.942	🔴	■ Category 0: Non-inhibitor; Category 1: inhibitor.<br>■ The output value is the probability of being inhibitor, within the range of 0 to 1.
BCRP inhibitor	0.09	🟢	■ Category 0: Non-inhibitor; Category 1: inhibitor.<br>■ The output value is the probability of being inhibitor, within the range of 0 to 1.
MRP1 inhibitor	0.888	🔴	■ Category 0: Non-inhibitor; Category 1: inhibitor.<br>■ The output value is the probability of being inhibitor, within the range of 0 to 1.

Table S4. Metabolism.

Property	Value	Decision	Comment
CYP1A2 inhibitor	0.0	🟢	■ Category 1: Inhibitor; Category 0: Non-inhibitor;<br>The output value is the probability of being inhibitor.
CYP1A2 substrate	0.015	🟢	■ Category 1: Substrate; Category 0: Non-substrate;<br>■ The output value is the probability of being substrate.
CYP2C19 inhibitor	0.022	🟢	■ Category 1: Inhibitor; Category 0: Non-inhibitor;<br>■ The output value is the probability of being inhibitor.
CYP2C19 substrate	0.794	🔴	■ Category 1: Substrate; Category 0: Non-substrate;<br>■ The output value is the probability of being substrate.
CYP2C9 inhibitor	0.442	🟠	■ Category 1: Inhibitor; Category 0: Non-inhibitor;<br>■ The output value is the probability of being inhibitor.

CYP2C9 substrate	0.109	●	■ Category 1: Substrate; Category 0: Non-substrate;<br>■ The output value is the probability of being substrate.
CYP2D6 inhibitor	0.068	●	■ Category 1: Inhibitor; Category 0: Non-inhibitor;<br>■ The output value is the probability of being inhibitor.
CYP2D6 substrate	0.0	●	■ Category 1: Substrate; Category 0: Non-substrate;<br>■ The output value is the probability of being substrate.
CYP3A4 inhibitor	0.872	●	■ Category 1: Inhibitor; Category 0: Non-inhibitor;<br>■ The output value is the probability of being inhibitor.
CYP3A4 substrate	0.734	●	■ Category 1: Substrate; Category 0: Non-substrate;<br>■ The output value is the probability of being substrate.
CYP2B6 inhibitor	0.472	●	■ Category 1: Inhibitor; Category 0: Non-inhibitor;<br>■ The output value is the probability of being inhibitor.
CYP2B6 substrate	0.001	●	■ Category 1: Substrate; Category 0: Non-substrate;<br>■ The output value is the probability of being substrate.
CYP2C8 inhibitor	0.763	●	■ Category 1: Inhibitor; Category 0: Non-inhibitor;<br>■ The output value is the probability of being inhibitor.
HLM Stability	0.778	●	■ human liver microsomal (HLM) stability<br>■ Category 0: stable+ (HLM > 30 min); Category 1: unstable- (HLM □ 30 min). The output value is the probability of human liver microsomal instability, where a value closer to 1 indicates a higher likelihood of instability. The range is between 0 and 1.

Table S5. Excretion.

Property	Value	Decision	Comment
CL_{plasma}	7.395	●	■ The unit of predicted CLplasma penetration is ml/min/kg. >15 ml/min/kg: high clearance; 5-15 ml/min/kg: moderate clearance; < 5 ml/min/kg: low clearance.
$T_{1/2}$	1.568	●	■ The unit of predicted T1/2 is hours.<br>■ ultra-short half-life drugs: 1/2 < 1 hour; short half-life drugs: T1/2 between 1-4 hours; intermediate short half-life drugs: T1/2 between 4-8 hours; long half-life drugs: T1/2 > 8 hours.

Table S6. Toxicity.

Property	Value	Decision	Comment
hERG Blockers	0.05	●	■ Molecules with IC50 □10□M or □50% inhibition at 10 □M were classified as hERG+ (Category 1),<br>■ while molecules with IC50 >10□M or < 50% inhibition at 10□M were classified as hERG - (Category 0).<br>■ The output value is the probability of being hERG+, within the range of 0 to 1.
hERG Blockers (10um)	0.31	●	■ Molecules with IC50 □10 □M are classified as hERG+ (Category 1),<br>■ and molecules with IC50 > 10□M are classified as hERG- (Category 0).<br>■ The output value is the probability of being hERG+, within the range of 0 to 1.
DILI	0.344	●	■ Drug Induced Liver Injury.<br>■ Category 1: drugs with a high risk of DILI;<br>■ Category 0: drugs with no risk of DILI.<br>■ The output value is the probability of being toxic.
AMES Muta genicity	0.803	●	■ AMES Toxicity<br>■ Category 1: Ames positive(+);<br>■ Category 0: Ames negative(-);<br>■ The output value is the probability of being toxic.
Rat Oral Acute Toxicity	0.653	●	■ Rat Oral Acute Toxicity.<br>■ Category 0: low-toxicity, > 500 mg/kg;<br>■ Category 1: high-toxicity; < 500 mg/kg.<br>■ The output value is the probability of being toxic, within the range of 0 to 1.
FDAMDD	0.69	●	■ FDA Maximum (Recommended) Daily Dose.<br>■ Category 1: FDAMDD (+);<br>■ Category 0: FDAMDD (-);<br>The output value is the probability of being positive.
Skin Sensiti zation	0.985	●	■ Category 1: Sensitizer;<br>■ Category 0: Non-sensitizer.<br>■ The output value is the probability of being toxic, within the range of 0 to 1.
Carcinogeni city	0.844	●	■ Category 1: carcinogens;<br>■ Category 0: non-carcinogens;<br>■ The output value is the probability of being toxic.
Eye Corrosion	0.372	●	■ Eye Corrosion<br>■ Category 1: corrosives;<br>Category 0: noncorrosives;<br>The output value is the probability of being corrosives.
Eye Irritation	0.949	●	■ Eye Irritation<br>■ Category 1: irritants;<br>Category 0: nonirritants;<br>The output value is the probability of being irritants.

Respiratory	0.539		■ Category 1: respiratory toxicants;<br>■ Category 0: non-respiratory toxicants.<br>The output value is the probability of being toxic, within the range of 0 to 1.
Human Hepatotoxicity	0.648		■ Human Hepatotoxicity<br>■ Category 1: H-HT positive(+);<br>■ Category 0: H-HT negative(-);<br>The output value is the probability of being toxic.
Drug-induced Nephrotoxicity	0.807		■ Category 0: non-nephrotoxic (-);<br>■ Category 1: nephrotoxic (+).<br>The output value is the probability of being nephrotoxic (+), within the range of 0 to 1.
Ototoxicity	0.253		■ Category 0: non-ototoxicity (-);<br>■ Category 1: ototoxicity (+).<br>The output value is the probability of being ototoxicity (+), within the range of 0 to 1.
Hematotoxicity	0.366		■ Category 0: non-hematotoxicity (-);<br>■ Category 1: hematotoxicity (+).<br>The output value is the probability of being hematotoxicity (+), within the range of 0 to 1.
Genotoxicity	0.692		■ Category 0: non-Genotoxicity (-);<br>■ Category 1: Genotoxicity (+).<br>The output value is the probability of being ototoxicity (+), within the range of 0 to 1.
RPMI-8226<br>Immunitoxicity	0.096		■ Category 0: non-cytotoxicity (-);<br>■ Category 1: cytotoxicity (+).<br>The output value is the probability of being ototoxicity (+), within the range of 0 to 1.
A549<br>Cytotoxicity	0.41		■ Category 0: non-cytotoxicity (-);<br>■ Category 1: cytotoxicity (+).<br>The output value is the probability of being ototoxicity (+), within the range of 0 to 1.
Hek293 Cytotoxicity	0.279		■ Category 0: non-cytotoxicity (-);<br>■ Category 1: cytotoxicity (+).<br>The output value is the probability of being ototoxicity (+), within the range of 0 to 1.
Drug-induced Neurotoxicity	0.701		■ Category 0: non-neurotoxic (-);<br>■ Category 1: neurotoxic (+).<br>The output value is the probability of being neurotoxic (+), within the range of 0 to 1.

5. ADMET evaluation of clitorin

The ADMET profile of clitorin was predicted using the following SMILES string: C[C@H]1[C@@H]([C@H]([C@H]([C@@H](O1)OC[C@@H]2[C@H]([C@@H]([C@H]([C@@H](O2)OC3=C(OC4=CC(=CC(=C4C3=O)O)O)C5=CC=C(C=C5)O)O[C

@H]6[C@@H]([C@@H]([C@H]([C@@H](O6)C)O)O)O)O)O)O)O)O. The complete prediction results are presented in Tables S7–S12. The color-coded decision indicators used throughout the tables are interpreted as follows: green, excellent; amber, medium; and red, poor.

Table S7. Physicochemical Property.

Property	Value	Comment
Molecular Weight	740.22	Contain hydrogen atoms. Optimal:100~600
Volume	673.908	Van der Waals volume
Density	1.098	Density = MW / Volume
nHA	19.0	Number of hydrogen bond acceptors. Optimal:0~12
nHD	11.0	Number of hydrogen bond donors. Optimal:0~7
nRot	8.0	Number of rotatable bonds. Optimal:0~11
nRing	6.0	Number of rings. Optimal:0~6
MaxRing	10.0	Number of atoms in the biggest ring. Optimal:0~18
nHet	19.0	Number of heteroatoms. Optimal:1~15
fChar	0.0	Formal charge. Optimal:-4 ~4
nRig	36.0	Number of rigid bonds. Optimal:0~30
Flexibility	0.222	Flexibility = nRot /nRig
Stereo Centers	15.0	Stereo Centers. Optimal: ≤ 2
TPSA	308.12	Topological Polar Surface Area. Optimal:0~140
logS	-2.416	The logarithm of aqueous solubility value.
logP	1.002	The logarithm of the n-octanol/water distribution coefficients at pH=7.4.
logD	1.467	The logarithm of the n-octanol/water distribution coefficient.
pka (Acid)	4.155	Acid-base dissociation constant (pKa) value represents the strength of a drug molecule's acidity or basicity.
pka (Base)	5.055	Acid-base dissociation constant (pKa) value represents the strength of a drug molecule's acidity or basicity.
Melting point	147.094	The predicted melting point of a compound is expressed in degrees Celsius (°C).<br>Melting points below 25°C are classified as liquids, while melting points above 25°C are classified as solids.
Boiling point	285.479	The predicted melting point of a compound is expressed in degrees Celsius (°C).<br>A normal boiling point below 25°C is categorized as a gas.

Table S8. Absorption.

Property	Value	Decision	Comment
Caco-2 Permeability	-6.655	●	Optimal: higher than -5.15 Log unit
MDCK Permeability	-4.973	●	■ low permeability: < 2 × 10-6 cm/s<br>■ medium permeability: 2-20 × 10^{-6} cm/s<br>■ high passive permeability: > 20 × 10^{-6} cm/s
PAMPA	1.0	●	■ The experimental data for Peff was logarithmically transformed (logPeff).<br>■ Molecules with log Peff values below 2.0 were classified as low-permeability (Category 0), while those with log Peff values exceeding 2.5 were classified as high-permeability (Category 1).
Pgp-inhibitor	0.0	●	■ Category 1: Inhibitor;<br>■ Category 0: Non-inhibitor;<br>■ The output value is the probability of being Pgp-inhibitor
Pgp-substrate	0.995	●	■ Category 1: substrate;<br>■ Category 0: Non-substrate;<br>■ The output value is the probability of being Pgp-substrate
HIA	0.136	●	■ Human Intestinal Absorption<br>■ Category 1: HIA+(HIA < 30%);<br>■ Category 0: HIA-(HIA >= 30%);<br>■ The output value is the probability of being HIA+
$F_{20\%}$	0.143	●	■ 20% Bioavailability<br>■ Category 1: F 20% + (bioavailability < 20%);<br>■ Category 0: F 20% - (bioavailability ≥ 20%);<br>■ The output value is the probability of being F 20% +
$F_{30\%}$	0.985	●	■ 30% Bioavailability<br>■ Category 1: F 30% + (bioavailability < 30%);<br>■ Category 0: F 30% - (bioavailability ≥ 30%);<br>■ The output value is the probability of being F 30% +
$F_{50\%}$	1.0	●	■ 50% Bioavailability<br>■ Category 1: F 50% + (bioavailability < 50%);<br>■ Category 0: F 50% - (bioavailability ≥ 50%);<br>■ The output value is the probability of being F 50% +

Table S9. Distribution.

Property	Value	Decision	Comment
PPB	81.997	●	■ Plasma Protein Binding<br>Optimal: < 90%.<br>■ Drugs with high protein-bound may have a low therapeutic index.
VDss	-0.079	●	■ Volume Distribution<br>■ Optimal: 0.04-20L/kg
BBB	0.0	●	■ Blood-Brain Barrier Penetration<br>■ Category 1: BBB+; Category 0: BBB-;<br>■ The output value is the probability of being BBB+
Fu	15.734	●	■ The fraction unbound in plasms<br>■ Low: <5%; Middle: 5~20%; High: > 20%
OATP1B1 inhibitor	0.999	●	■ Category 0: Non-inhibitor; Category 1: inhibitor.<br>■ The output value is the probability of being inhibitor, within the range of 0 to 1.
OATP1B3 inhibitor	1.0	●	■ Category 0: Non-inhibitor; Category 1: inhibitor.<br>■ The output value is the probability of being inhibitor, within the range of 0 to 1.
BCRP inhibitor	0.343	●	■ Category 0: Non-inhibitor; Category 1: inhibitor.<br>■ The output value is the probability of being inhibitor, within the range of 0 to 1.
MRP1 inhibitor	0.006	●	■ Category 0: Non-inhibitor; Category 1: inhibitor.<br>■ The output value is the probability of being inhibitor, within the range of 0 to 1.

Table S10. Metabolism.

Property	Value	Decision	Comment
CYP1A2 inhibitor	0.0	●	■ Category 1: Inhibitor; Category 0: Non-inhibitor;<br>The output value is the probability of being inhibitor.
CYP1A2 substrate	0.0	●	■ Category 1: Substrate; Category 0: Non-substrate;<br>■ The output value is the probability of being substrate.
CYP2C19 inhibitor	0.0	●	■ Category 1: Inhibitor; Category 0: Non-inhibitor;<br>■ The output value is the probability of being inhibitor.
CYP2C19 substrate	0.0	●	■ Category 1: Substrate; Category 0: Non-substrate;<br>■ The output value is the probability of being substrate.

CYP2C9 inhibitor	0.0	●	■ Category 1: Inhibitor; Category 0: Non-inhibitor;<br>■ The output value is the probability of being inhibitor.
CYP2C9 substrate	0.0	●	■ Category 1: Substrate; Category 0: Non-substrate;<br>■ The output value is the probability of being substrate.
CYP2D6 inhibitor	0.0	●	■ Category 1: Inhibitor; Category 0: Non-inhibitor;<br>■ The output value is the probability of being inhibitor.
CYP2D6 substrate	0.0	●	■ Category 1: Substrate; Category 0: Non-substrate;<br>■ The output value is the probability of being substrate.
CYP3A4 inhibitor	0.004	●	■ Category 1: Inhibitor; Category 0: Non-inhibitor;<br>■ The output value is the probability of being inhibitor.
CYP3A4 substrate	0.0	●	■ Category 1: Substrate; Category 0: Non-substrate;<br>■ The output value is the probability of being substrate.
CYP2B6 inhibitor	0.0	●	■ Category 1: Inhibitor; Category 0: Non-inhibitor;<br>■ The output value is the probability of being inhibitor.
CYP2B6 substrate	0.0	●	■ Category 1: Substrate; Category 0: Non-substrate;<br>■ The output value is the probability of being substrate.
CYP2C8 inhibitor	1.0	●	■ Category 1: Inhibitor; Category 0: Non-inhibitor;<br>■ The output value is the probability of being inhibitor.
HLM Stability	0.056	●	■ human liver microsomal (HLM) stability<br>■ Category 0: stable+ (HLM > 30 min); Category 1: unstable- (HLM ≤ 30 min). The output value is the probability of human liver microsomal instability, where a value closer to 1 indicates a higher likelihood of instability. The range is between 0 and 1.

Table S11. Excretion.

Property	Value	Decision	Comment
CL_{plasma}	0.981	●	■ The unit of predicted CLplasma penetration is ml/min/kg. >15 ml/min/kg: high clearance; 5-15 ml/min/kg: moderate clearance; < 5 ml/min/kg: low clearance.
$T_{1/2}$	4.962	●	■ The unit of predicted T1/2 is hours.<br>■ ultra-short half-life drugs: 1/2 < 1 hour; short half-life drugs: T1/2 between 1-4 hours; intermediate short half-life drugs: T1/2 between 4-8 hours; long half-life drugs: T1/2 > 8 hours.

Table S12. Toxicity.

**Property**	**Value**	**Decision**	**Comment**
hERG Blockers	0.01	●	■ Molecules with IC50 ≤10μM or ≥50% inhibition at 10 μM were classified as hERG+ (Category 1),<br>■ while molecules with IC50 >10μM or < 50% inhibition at 10μM were classified as hERG - (Category 0).<br>■ The output value is the probability of being hERG+, within the range of 0 to 1.
hERG Blockers (10um)	0.201	●	■ Molecules with IC50 ≤10 μM are classified as hERG+ (Category 1),<br>■ and molecules with IC50 > 10μM are classified as hERG- (Category 0).<br>■ The output value is the probability of being hERG+, within the range of 0 to 1.
DILI	0.877	●	■ Drug Induced Liver Injury.<br>■ Category 1: drugs with a high risk of DILI;<br>■ Category 0: drugs with no risk of DILI.<br>■ The output value is the probability of being toxic.
AMES Muta genicity	0.738	●	■ AMES Toxicity<br>■ Category 1: Ames positive(+);<br>■ Category 0: Ames negative(-);<br>■ The output value is the probability of being toxic.
Rat Oral Acute Toxicity	0.01	●	■ Rat Oral Acute Toxicity.<br>■ Category 0: low-toxicity, > 500 mg/kg;<br>■ Category 1: high-toxicity; < 500 mg/kg.<br>■ The output value is the probability of being toxic, within the range of 0 to 1.
FDAMDD	0.03	●	■ FDA Maximum (Recommended) Daily Dose.<br>■ Category 1: FDAMDD (+);<br>■ Category 0: FDAMDD (-);<br>The output value is the probability of being positive.
Skin Sensiti zation	0.99	●	■ Category 1: Sensitizer;<br>■ Category 0: Non-sensitizer.<br>■ The output value is the probability of being toxic, within the range of 0 to 1.
Carcinogeni city	0.021	●	■ Category 1: carcinogens;<br>■ Category 0: non-carcinogens;<br>■ The output value is the probability of being toxic.
Eye Corrosion	0.0	●	■ Eye Corrosion<br>■ Category 1: corrosives;<br>Category 0: noncorrosives;<br>The output value is the probability of being corrosives.

Eye Irritation	0.432		■ Eye Irritation<br>■ Category 1: irritants;<br>Category 0: nonirritants;<br>The output value is the probability of being irritants.
Respiratory	0.003		■ Category 1: respiratory toxicants;<br>■ Category 0: non-respiratory toxicants.<br>The output value is the probability of being toxic, within the range of 0 to 1.
Human Hep atotoxicity	0.313		■ Human Hepatotoxicity<br>■ Category 1: H-HT positive(+);<br>■ Category 0: H-HT negative(-);<br>The output value is the probability of being toxic.
Drug-induce d Nephrotox icity	0.249		■ Category 0: non-nephrotoxic (-);<br>■ Category 1: nephrotoxic (+).<br>The output value is the probability of being nephrotoxic (+), within the range of 0 to 1.
Ototoxicity	0.951		■ Category 0: non-ototoxicity (-);<br>■ Category 1: ototoxicity (+).<br>The output value is the probability of being ototoxicity (+), within the range of 0 to 1.
Hematotoxic ity	0.03		■ Category 0: non-hematotoxicity (-);<br>■ Category 1: hematotoxicity (+).<br>The output value is the probability of being hematotoxicity (+), within the range of 0 to 1.
Genotoxicity	0.744		■ Category 0: non-Genotoxicity (-);<br>■ Category 1: Genotoxicity (+).<br>The output value is the probability of being ototoxicity (+), within the range of 0 to 1.
RPMI-8226<br>Immunitoxici ty	0.167		■ Category 0: non-cytotoxicity (-);<br>■ Category 1: cytotoxicity (+).<br>The output value is the probability of being ototoxicity (+), within the range of 0 to 1.
A549<br>Cytotoxicity	0.464		■ Category 0: non-cytotoxicity (-);<br>■ Category 1: cytotoxicity (+).<br>The output value is the probability of being ototoxicity (+), within the range of 0 to 1.
Hek293 Cytotoxicity	0.704		■ Category 0: non-cytotoxicity (-);<br>■ Category 1: cytotoxicity (+).<br>The output value is the probability of being ototoxicity (+), within the range of 0 to 1.
Drug-induce d Neurotoxi city	0.0		■ Category 0: non-neurotoxic (-);<br>■ Category 1: neurotoxic (+).<br>The output value is the probability of being neurotoxic (+), within the range of 0 to 1.
